# Meta-analysis of 633,317 individuals shows associations between healthy diets and depression, anxiety and stress in 23 low- and middle-income countries

**DOI:** 10.1186/s44263-026-00283-w

**Published:** 2026-06-01

**Authors:** Thalia M. Sparling, Cesar Cornejo, Bryan Cheng, Lisa M. Troy, Suneetha Kadiyala

**Affiliations:** 1 London School of Hygiene and Tropical Medicine, Keppel Street, London, WC1E 7HT UK; 2 Global Mental Health Lab, Teachers College, Columbia University, New York, NY USA; 3 School of Public Health and Health Sciences, University of Massachusetts, Amherst, MA USA

**Keywords:** Depression, Anxiety, Stress, Common mental disorders, Food intake, Dietary patterns, Systematic review, Multi-level meta-analysis

## Abstract

**Background:**

Poor diet quality related to common mental disorders contribute to global health syndemics. However, there is no synthesis quantifying associations specifically in Low- and Middle-Income Countries (LMIC) where these concomitant health burdens are most prevalent.

**Methods:**

We drew on a systematic Evidence and Gap Map (EGM) of > 3,000 records from Medline, CAB Global Health and PsycINFO (2000–2024). We selected LMIC studies quantifying healthy diets (validated dietary indices or factor-analytic methods) against validated screening measures of depression, anxiety, and stress, with a healthy versus unhealthy diet comparator. Effect sizes were standardised as mean differences from Hedges’ g and pooled using three-level meta-analysis with robust variance estimation (RVE). Risk of bias was assessed, and sensitivity analyses showed robustness across study designs, dietary measures, and country income strata.

**Results:**

Eighty-three eligible studies from 23 countries (depression *n* = 69; anxiety *n* = 43; stress *n* = 26), and 65 LMIC sample populations, reported statistical measures for 633,317 unique individuals. The Standardized Mean Differences (SMD) comparing healthy diets to unhealthy diets were -0.29 for depression (95% CI -0.35 to -0.23), -0.25 for anxiety (95% CI -0.35 to -0.16), and -0.24 for stress (95% CI -0.33 to -0.14). Results remained robust when restricted to low Risk of Bias studies. Findings were similar in direction and magnitude across study designs, dietary measurements, diagnostic tools, country income levels, and estimates adjusted for socio-economic status. Methodological limitations (e.g., cross-sectional design) and few studies from low-income countries created evidence gaps.

**Conclusions:**

Healthy diets were consistently associated with lower depression, anxiety, and stress symptoms in LMIC. These findings call for integrated dietary and mental health programming in LMIC (and in any setting with disproportionate health vulnerabilities), and for longitudinal and intervention research across diverse low-income settings beyond Iran and China.

**Supplementary Information:**

The online version contains supplementary material available at 10.1186/s44263-026-00283-w.

## Background

We are living in an era of global health syndemics, where multiple diseases interact synergistically within populations under conditions of social and structural inequity [1]. The most vulnerable people bear concomitant health burdens, compounding their health risk overall. Common mental health disorders (CMDs) such as depression and anxiety, contributed 17.2% of life years lost to disability in 2021 [2], with higher prevalence of CMDs reported in Low- and Middle-Income Countries (LMIC) [3]. CMDs are drivers and consequences of other marginalisations, such as food insecurity and unhealthy diet. In turn, 35% of the global population could not afford a healthy diet in 2022. Of those, 1.68 billion (nearly 60%) live in LMIC [4], where on average only 2.1% of government health expenditure goes to mental health [5] and public spending on the food systems that drive dietary trends is both low (e.g., less than $10 expenditure on agriculture per rural inhabitant in Low Income Countries) and stagnant [4]. This is especially problematic since food insecurity and malnutrition are most prevalent in these settings.

In LMIC, CMDs are almost always measured through non-specialist administered screening tools [6]. Many screening tools were developed and validated to align with diagnostic criteria, for instance the Diagnostic and Statistical Manual of Mental Disorders (DSM) [7]. However, in epidemiological research, these tools can feasibly be used for defining cases where tools have been validated for cut-offs corresponding to diagnostic criteria, or for measuring symptomatology through continuous measures [8–11]. This helps focus on the range and extent of common mental health symptoms in diverse populations, and the implications of poor mental health, such as functional loss or wider impacts on health indices [12–15]^,^.

A healthy diet consists of balanced proportions (enough to meet caloric adequacy but not exceed it) of primarily whole grains, vegetables, fruit, nuts, seeds, and where available fish, while moderating meat and dairy, and limiting consumption of sugars, saturated fats, and ultra-processed foods (UPFs) [16]. In simpler terms, a healthy diet is health-promoting and disease-preventing [17]. Healthy diets are measured through many different instruments, including adherence to (national) dietary recommendations, adherence to certain diets (e.g. the mediterranean diet (MD) or the dietary inflammatory index (DII)), scoring or counts of food items grouped to indicate dietary diversity and/or certain index foods that are considered beneficial to health (e.g. dietary diversity of women, children or households). Another common measure in nutritional epidemiology is an a priori statistical method of Principal Components Analysis (PCA), which groups intakes into consumption or diet patterns [18]. These indices and measures are validated similarly in that they correspond to increased probability of nutrient adequacy (at least in aggregate at the population level).

Healthy diets resulting in better mental health is plausible from many physiological and social arguments. Potential biological pathways related to mental disorders include inflammation, oxidative stress, the gut microbiome, brain plasticity, mitochondrial dysfunction, epigenetics, and the Hypothalamic-Pituitary-Adrenal (HPA) axis, among others – factors which can interact and follow multiple pathways [19]. Evidence shows that healthy dietary patterns can beneficially modulate each of these interconnected systems [20, 21]. For instance, healthy diets are usually considered more diverse, containing higher quantities of foods that contain antioxidants (e.g., polyphenols such as flavones and anthocyanins), long-chain fatty acids (e.g., eicosapentaenoic acid and docosahexaenoic acid), and other essential nutrients that lower risk of nutrition-related chronic disease (e.g., obesity, diabetes, cancer) [16]. These and other dietary components reduce inflammation [20, 22–24]. Diet can also improve neurotransmission and neurocognitive function through enhanced brain plasticity via brain-derived neurotrophic factor (BDNF) [25]. Higher socioeconomic status, wealth equity and better lifestyle factors may also underpin both healthier eating and better mental health [26].

In the reverse direction, poor mental health can impact diet quality through multiple pathways. Depression and anxiety are associated with diminished motivation, cognitive impairments, and reduced executive function [27, 28], all of which can hinder meal planning, food shopping and preparation [29]. People experiencing poor mental health may also have altered appetite regulation and food preferences, leading to consumption of energy dense and UPFs [30], although evidence now more strongly points to UPF consumption as a risk factor for depression [31]. The impact of mental health on diet can extend beyond the individual to the household level, as studies have shown that children of parents, particularly mothers, with CMDs are more likely to experience worse diet quality and child feeding outcomes [32–34].

We have limited evidence about the proportion of the world’s population who simultaneously experience poor mental health and poor diet quality [35]. However, research shows that these burdens are interlinked, and can exacerbate one another [36]. Some longitudinal and review studies show that healthy diets improve mental health [37–44], including in adolescents [45–48]. Some cohort studies examining bidirectional relationships also show that better mental health can also contribute to eating healthier diets [49]. Meta-analyses of dietary intervention trials support causal evidence for impacts on mental health: one meta-analysis on all dietary interventions found impacts on depression (especially for women), although no overall effect on anxiety [50], while Mediterranean Diet trials among those already with depression showed reduced depressive symptoms [51].

Previous systematic reviews on this topic have examined depression alone [44, 52, 53], focused only on a few dietary indices [53, 54], or were umbrella reviews without pooled estimates [52, 53]. Most importantly, these reviews do not disaggregate their analysis by geographic or income setting. There is no stand-alone meta-analysis about the interplay between diet and mental health in LMIC, a critical gap given that these relationships may differ in these settings, and moreover that they host higher burdens of both poor diet quality and symptoms of CMDs. From an Evidence and Gap Map including all aspects of food security and nutrition related to depression, anxiety, stress and wellbeing [55], we used reports from populations living in LMIC that quantify healthy diets measured against depression, anxiety and stress measures. We pool their effect estimates to assess their robustness and identify evidence gaps.

## Methods

The selection of studies and data extraction for this analysis consisted of 2 steps. We first relied on the inclusion and exclusion criteria, screening process, and data extraction from a large systematic Evidence and Gap Map (EGM) that included peer-reviewed, English language studies linking food security and nutrition measures to common mental health problems (depression, anxiety, stress and mental wellbeing) in the general population, published from January 1, 2000 to January 31, 2024, following PRISMA Extension for Scoping Reviews (PRISMA-ScR) reporting consistent with evidence mapping guidelines [56]. For the EGM, we screened 30,896 records published from 2000 through June 2020 for the original EGM, resulting in a map and analysis of 1945 studies [57]. To update the EGM, we screened 13,490 additional records published from 2020 to January 2024 and included 1,107 additional records [55] using the same search both times with a one-year overlap to account for indexing lags (Supplementary Material 1: Table S1). The updated EGM includes over 3,000 studies on this topic.

**Fig. 1:**
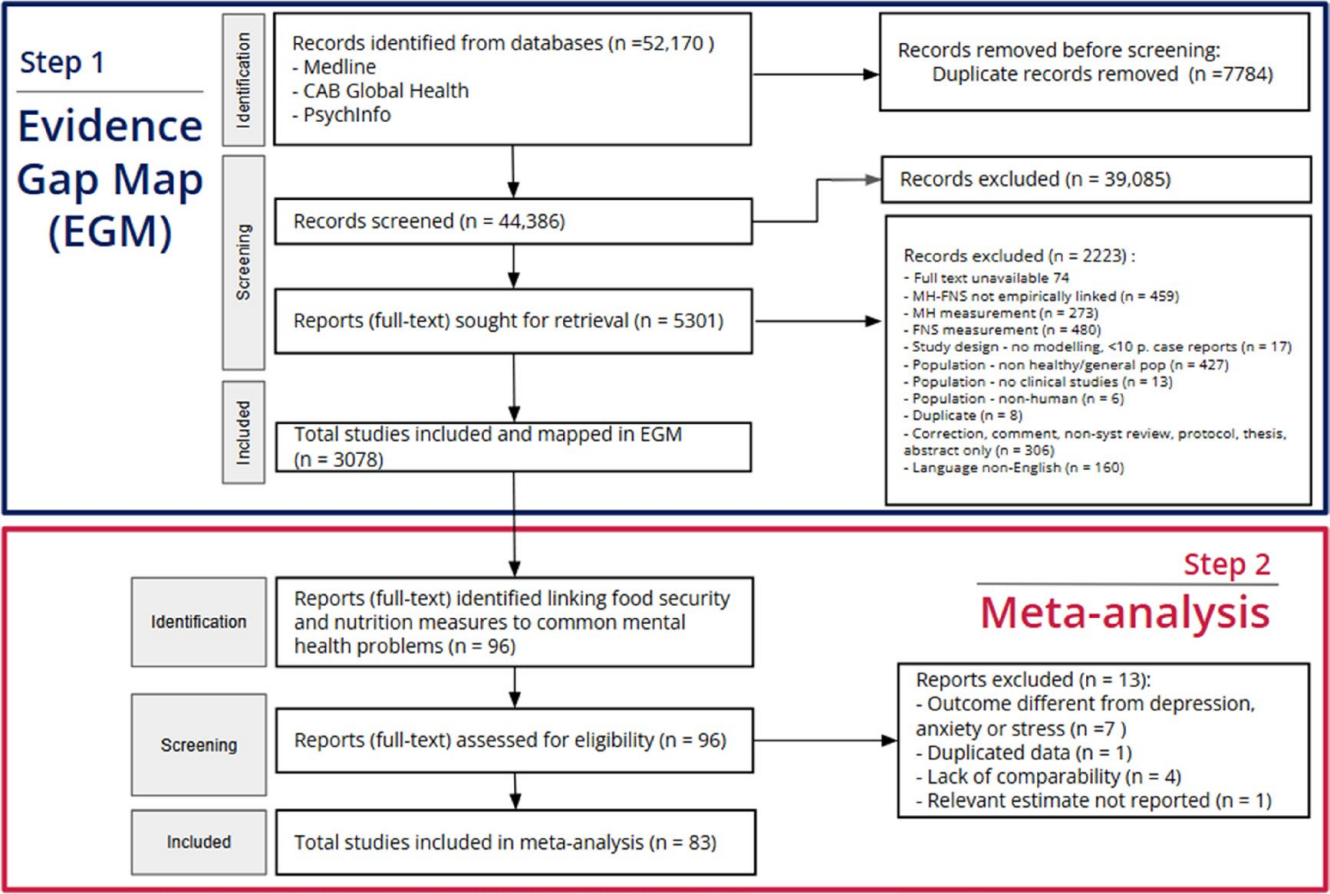
PRISMA diagram. Adapted Preferred Reporting Items for Systematic Reviews and Meta-Analyses (PRISMA) flow chart representing the 2-step study selection process. Step 1 reports evidence gap map studies at the identification, screening and final inclusion stages. Step 2 reports the meta-analysis studies at each stage, which started by identifying eligible studies included in the evidence gap map. MH: mental health. FNS: food and nutrition security

Drawing on the EGM repository (a summary PRISMA flowchart is provided for this selection, Fig. 1) [58], we selected a subset of studies for the meta-analysis, reporting according to the Meta-analysis Of Observational Studies in Epidemiology (MOOSE) guidelines (checklist in Supplementary Material 1: Table S2) [59]. For healthy diets, we selected all studies fitting the eligibility criteria of the EGM, which were independently screened by two reviewers, with additional eligibility review by a senior researcher. All studies in the EGM were coded based on specific measures and indicators used, first by a single researcher, then through iterative full-record and domain-specific checks by a senior researcher. Then, aligning with the broad definition of healthy diets proposed by Cena and Calder [16], we used relevant measures from the ‘diets’ domain of the EGM that would capture ‘healthy’ diets, such as the Healthy Eating Index [60] (HEI) and its iterations [61], dietary diversity [62], the DII [63], and dietary patterns identified through factor analysis or clustering of foods measured through intake questionnaires (e.g., Food Frequency Questionnaire (FFQ)) [64]. A full list of all diet measures is provided in Table 1.

**Table 1:** Distribution of healthy diet measures and mental health diagnostic tools. Dietary measures were grouped into 5 groups. Mental health measures are presented per outcome, but each tool can measure more than one outcome

Groups	Dietary measurements	Number of studies^1^
Group 1: Adherence and adequacy (G1)^2^	- Adherence to diet recommendations - Nutrient adequacy	2
Group 2: Dietary patterns reducing nutrition-related chronic diseases (G2) ^2^	- Dietary Approaches to Stop Hypertension (DASH) - Dietary Inflammatory Index - Mediterranean diet	22
Groups 3: Diet diversity indices (G3) ^2^	- Dietary diversity (e.g., Minimum Dietary Diversity of Women, Individual Dietary Diversity Score) - Any other Dietary Variety Scores	15
Group 4: Diet quality indices (G4) ^2^	- Diet Quality Indexes (all) - Global Diet Quality Score - Global Dietary Index - Healthy Eating Indexes (all)	19
Group 5: Factor analysis and others (G5) ^3^	All comparisons between a healthy and an unhealthy diet. Unhealthy is a group with a processed, western, modern, traditional, or unhealthy diet determined for each study.	26
**Mental Health Outcome (number of studies** ^**4**^ **)**	**Validated diagnostic tool**	**Number of studies** ^**4**^
Depression (69)	Depression, Anxiety and Stress Scale (DASS)	14
	Depression subscale of the Hospital Anxiety and Depression Scale (HADS-D)	11
	Patient Health Questionnaire (PHQ-9)	11
	Beck Depression Inventory (BDI)	7
	Center for Epidemiological Studies - Depression scale (CES-D)	7
	Edinburgh Postpartum Depression Scale (EPDS)	7
	Clinical/diagnostic interview (CIDI - SF)	2
	Geriatric Depression Scale (GDS)	2
	Mini International Neuropsychiatric Interview (MINI)	2
	Zung self-rating scale	2
	6-item Kutcher Adolescent Depression Scale (KADS-6)	1
	Multidimensional Sub-health Questionnaire of Adolescents (MSQA)	1
	Primary Care Evaluation of Mental Disorders (PRIME-MD)	1
	Self-Rating Depression Scale (SDS)	1
Anxiety (43)	Depression, Anxiety and Stress Scale (DASS)	15
	Hospital Anxiety and Depression Scale (HADS-A)	11
	General Anxiety Disorder Scale (GAD)	10
	Coronavirus Anxiety Scale (CAS)	1
	Mini International Neuropsychiatric Interview (MINI)	1
	Multidimensional Sub-health Questionnaire of Adolescents (MSQA)	1
	Primary Care Evaluation of Mental Disorders (PRIME-MD)	1
	Zung Self-reported Anxiety Scale (Zung SAS)	1
	State-Trait Anxiety Inventory (STAI)	1
Stress (26)	Depression, Anxiety and Stress Scale (DASS)	14
	General Health Questionnaire (GHQ)	9
	Perceived Stress Scale (PSS)	3

1 One study measured diets with both a Group 2 and a Group 4 measurement, so it is double-counted. 2 A priori measure. 3 A posterior measure. 4 Several studies reported 2 or 3 mental health outcomes, so the count exceeds the total number of studies (n = 83). Grouping of tools into “depression”, “anxiety” and “stress” was based on which mental health outcome they were used to measure in included studies, not according to the defined outcome of the tool itself

Among the mental health domains in the EGM, we included all the studies that measured depression, anxiety or stress using any validated tool, such as mental health screening instruments like the Centre for Epidemiological Studies – Depression Scale (CES-D), the Depression, Anxiety and Stress Scale (DASS), the Generalised Anxiety and Depression Scale (GAD) or the State Trait Anxiety Index (STAI). In this second step, our exclusion criteria for the meta-analysis consisted of (1) Mental health different from depression, anxiety or stress, (2) Lack of comparability of diet measurements (not valid or comparable within the meta-analysis), (3) Duplicated data: same sample and same results of an already included study, and (4) Relevant estimate not reported. We excluded any study with a measure of healthy diet without an unhealthy diet comparator. The detailed list of inclusion and exclusion criteria for the EGM and the meta-analysis, including all the diet and MH measurements out of scope are detailed in the Supplementary Material 1: Table S3.

We grouped eligible studies according to the dietary measurements used (see Table 1 in Results). Group 1 included adherence and adequacy adherence to diet recommendations and nutrient adequacy. Group 2 included dietary patterns shown to reduce nutrition-related chronic diseases, such as Dietary Approaches to Stop Hypertension (DASH), DII, and MD. Group 3 was made up of diet diversity indices like Minimum Dietary Diversity of Women (MDD-W) and Individual Dietary Diversity Score (IDDS) as well as any other dietary variety scores. Group 4 included all Diet Quality Indices, Global Diet Quality Score (GDQS), Global Dietary Index (GDI), and all versions of the HEI. Groups (1–4) are all measured via predefined patterns and/or specific food intake (*a priori* methods), thus the observed dietary pattern in the population is compared to a preexisting index. Group 5 included any measure derived from factor analysis, Principal Component Analysis (PCA) or other data-driven (*a posteriori*) approaches, which comprise comparisons between clustered patterns of food intake in a study population (usually between a ‘healthy pattern’ and an ‘unhealthy’ one, e.g., processed, western, modern, or unhealthy).

For included studies, two researchers independently extracted data on effect sizes (since most of the descriptive characteristics of studies were already coded in the EGM), along with claim type (associational or causal), exposure scale (healthy or unhealthy), sample size, number of women, percentage of women in the sample, age group, mean age, age standard deviation, and reported statistical measures. Specifically, we extracted the following statistical effect sizes: Beta Coefficients (β), Odds Ratio (OR), Adjusted Odds Ratio (AOR), Risk Ratio (RR), Hazard Ratio (HR), Pearson’s Correlation Coefficient (r), Mean Differences, adjusted or non-adjusted estimations, socio-economic factors covariates. We made sure to capture the direction of the ‘healthy’ effect on mental health symptoms.

Effect sizes were converted to standardized metrics to ensure comparability across studies. For odds ratios (OR), risk ratios (RR), and prevalence ratios (PR), the Chinn transformation [65] was applied to obtain Cohen’s d. β coefficients were standardized using the pooled standard deviation, while mean differences were converted by dividing the difference between group means by the pooled standard deviation. Hazard ratios were adjusted based on their distribution properties. To correct for small sample bias, Hedges’ g was computed [66]. Pearson’s r was derived from standardized mean differences, and Fisher’s z transformation was applied to normalize correlations. As the primary effect size index, we selected the Standardized Mean Difference (SMD) estimated by Hedges’ g due to its robustness in meta-analyses, allowing for comparisons of means and regression coefficients across diverse study designs [67], and easier interpretability [66].

When studies used data from the same sample population, but reported a different sample size, we extracted the highest number to estimate sample populations. To estimate pooled effect sizes, we conducted a three-level meta-analysis using a robust variance estimation (RVE) framework. The three-level meta-analysis dealt with the dependency coming from studies drawing estimates from the same populations [68], and we modelled our data to account for differences at the individual effect sizes, within sample population and between sample populations. The RVE allows adjustment for the standard errors and improves the statistical inferences when we face a data dependency issue [69]. The model was estimated using the restricted maximum-likelihood (REML) estimator [70], incorporating nested random effects at the study and effect-size levels. Variance components were estimated using a structured variance-covariance matrix with a predefined correlation coefficient (ρ = 0.5) to model dependence among estimates derived from the same dataset. Observations were weighted by their inverse variance, and heterogeneity was assessed through variance decomposition (I²) across levels. We considered both confidence intervals and prediction intervals, the latter of which is a more transparent indicator of heterogeneity at a population (vs. sample) level. To evaluate the robustness of the findings, sensitivity analyses included alternative specifications of the correlation parameter, exclusion of influential studies, and comparisons across effect size metrics (Hedges’ g, Cohen’s d, Fisher’s z).

Outlier and influential study detection were performed using Studentized residuals and Cook’s distances. Studies were flagged as potential outliers if their Studentized residual exceeded the 100 × (1–0.05/ (2 × k))th percentile of a standard normal distribution, applying a Bonferroni correction for multiple comparisons with a two-sided α = 0.05 across k studies. Influential studies were identified using Cook’s distance, where values exceeding the median plus six times the interquartile range (IQR) were considered indicative of undue influence on model estimates.

We tested alternative approaches to deal with the dependency of the data [71]. Model comparison was conducted by testing a reduced two-level model (removing the second random effect) against the full three-level specification to assess whether modelling within-study dependence significantly improved model fit [72]. The full three-level model yielded lower Akaike Information Criterion (AIC), Bayesian Information Criterion (BIC), and corrected AIC (AICc) values, alongside a higher log-likelihood (logLik), indicating a superior fit [73]. The likelihood ratio test (LRT) comparing the two models returned a p-value of 0.0831, suggesting that while the improvement in fit was not statistically significant at the conventional 5% level, it was marginal. Given the hierarchical nature of the data, where multiple effect sizes stem from the same study population, the three-level model was retained as the preferred approach to appropriately account for dependency and reduce bias in pooled estimates [72].

For consistency, the same senior researcher assessed risk of bias (RoB) for all studies using the “Quality In Prognosis Studies” (QUIPS) tool (Supplementary Material 1: Table S4) [74]. In addition, 12% of the studies were assessed by two reviewers independently, and an additional 10% of studies were reviewed by a second researcher. Although the QUIPS tool is designed for prognostic studies, prognoses are similar to risks in epidemiology. Furthermore, most of the included studies are cross-sectional, and there is no existing tool for these that covers all important domains of possible bias [75]. The QUIPS covers many of the important domains identified, including study participation, study attrition (omitted for cross-sectional studies), prognostic factor (exchanged for exposure) measurement, outcome measurement, study confounding, and statistical analysis and reporting. For each of these domains, studies were ranked as having low, moderate or high risk of bias. Then, we grouped all studies in 2 groups: high RoB (has at least 1 domain with high RoB) and low RoB (no domain has high RoB).

We examined the publication trends over time; used the country-level income classification as defined by the World Bank at the time of this analysis (2024) [76] to examine effects by low-, lower-middle, and upper-middle income country status; and analysed the geographic spread across regions and countries. We included literature where healthy diets and mental health were associated, including when the hypothesised exposure was healthy diets or mental health, and vice versa for the outcome or dependent variable. We used the ‘hypothesis direction’ classification in the EGM to examine these different groups, as well as analysing potentially differential effects for different population groups (e.g. adults, elderly, adolescents, or parents paired with their children). We examined pooled estimates by dietary measure group (Table 1).

We conducted several sensitivity analyses by different study characteristics: study design, dietary measurements and country-level income classification. We also ran an analysis of subgroups of studies based on whether they adjusted for socio-economic status (SES) factors, presenting pooled estimates from studies with adjustment for SES, those without, and comparing to the overall. All our sensitivity analyses were conducted first on the entire set of studies, then restricting to low RoB studies, and restricting to the high RoB studies.

## Results

### Study selection

From the previous Evidence and Gap Map (EGM) (Summarised in step 1, Fig. 1), we systematically included a subset of studies for the meta-analysis. A total of 96 studies met inclusion criteria from the EGM, from which we excluded 13 studies: 7 had a mental health outcome different from depression, anxiety or stress (e.g., common mental disorders and mental wellbeing), 1 reported the same data and results in another included study, 4 lacked comparability (e.g., did not define healthy vs. unhealthy diet patterns), 1 did not have relevant data reported (i.e., only percentiles) from which it is not possible to calculate comparable statistics.

### Sample populations

Eighty-three studies were included (depression = 69; anxiety = 43; stress = 26), from which we extracted 139 effect estimates (depression = 70; anxiety = 43; stress = 26) from 65 unique sample populations (depression = 58; anxiety = 31; stress = 20). These estimates were pooled from 633,317 unique individuals (depression = 473,236; anxiety = 146,217; stress = 24,690). The most studied populations were adults (*n* = 42), followed distantly by females (*n* = 16), mid- to later-life populations (*n* = 12), pregnant women or mothers (*n* = 10), and adolescents (*n* = 7). The list of included studies is presented in Supplementary Material 1: Table S5.

### Sample settings

**Fig. 2:**
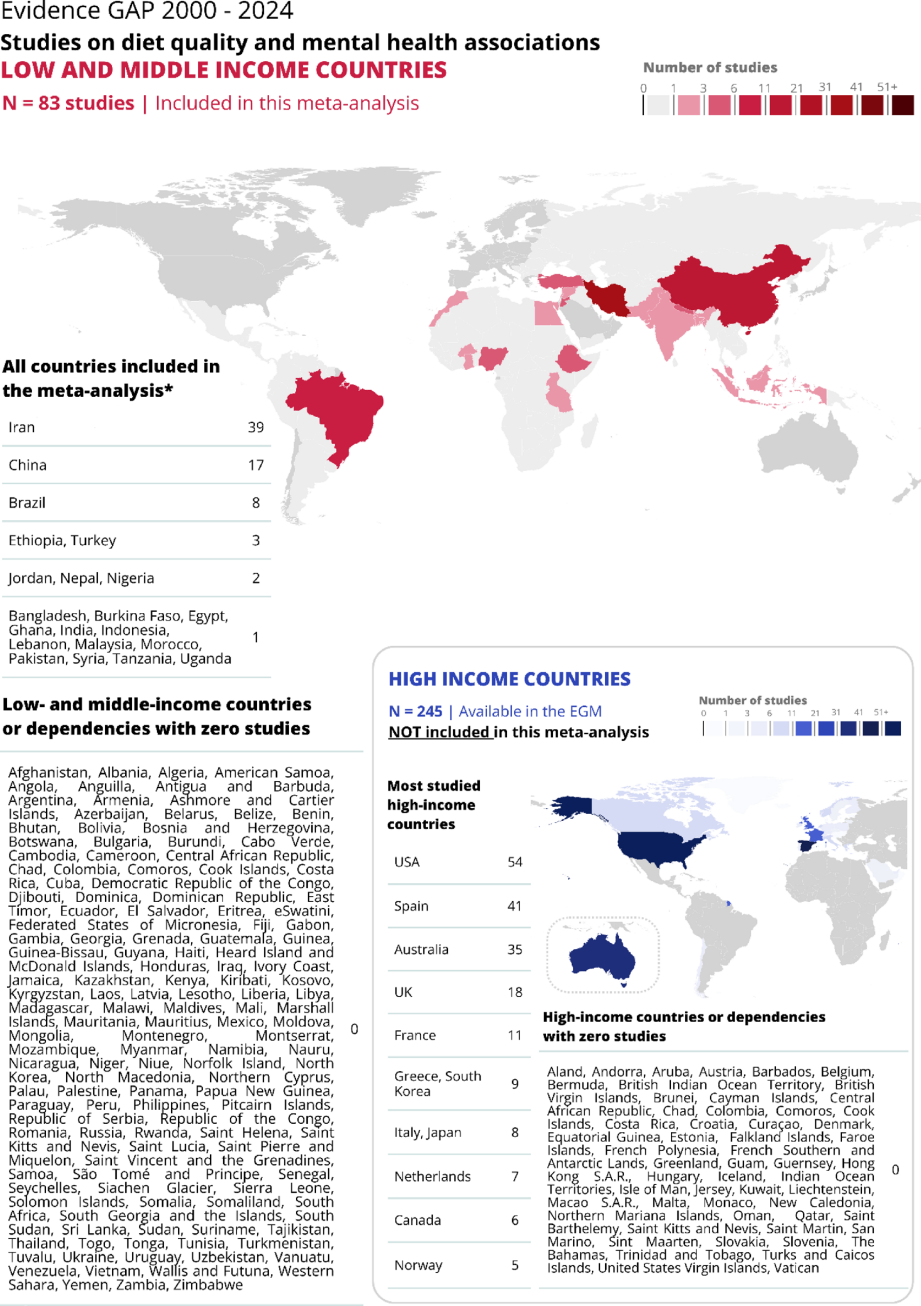
Geographic and country income-level distribution of studies reporting associations between healthy diets and mental health symptoms. For comparison, the graph includes high income studies (in blue, *n* = 245) alongside LMIC (in red, *n* = 89). The high-income studies are not included in this meta-analysis but are available in the EGM. *Two studies cover more than one country, thus there are 83 studies included and 89 countries studied.

The 83 studies covered 23 countries: 4 low-income, 11 lower-middle income and 69 upper-middle income (Fig. 2), where 1 study was across six countries, 3 of which were low-income and 3 were low-middle income. Most studies were based in Asia (*n* = 70), of which 39 were from Iran and 17 were from China (both upper-middle income countries). The rest of Asian countries studied were Turkey, Jordan, Nepal, Bangladesh, and India. Outside of Asia, 8 studies were from South America (all from Brazil) and 6 from Africa (3 of which were from Ethiopia). The low-income country studies were from Burkina Faso, Ethiopia, Lebanon, Syria and Uganda. Although we only include results from LMIC in this meta-analysis, we identified 245 additional studies from high-income countries from the EGM, which are included for comparative purposes in Fig. 2. There were 188 administrative units (out of 241 aggregated territories defined by the Natural Earth map dataset version 5.1.1) world-wide where there was not a single study on this topic.

### Study characteristics

There were no published studies on this topic between 2000 and 2012 and then there was a steady annual increase from 1 in 2013 to 15 in 2023. The primary study design was cross-sectional (*n* = 71). The rest were longitudinal (*n* = 9), and case-control (*n* = 3). We classified the proposed direction of association in studies, regardless of design (i.e., diet as an exposure for mental health outcomes and vice versa). Most (*n* = 73) framed their research as studying the effect of diets on mental health symptoms, and 10 declared studying the effect of mental health symptoms on diets.

**Fig. 3:**
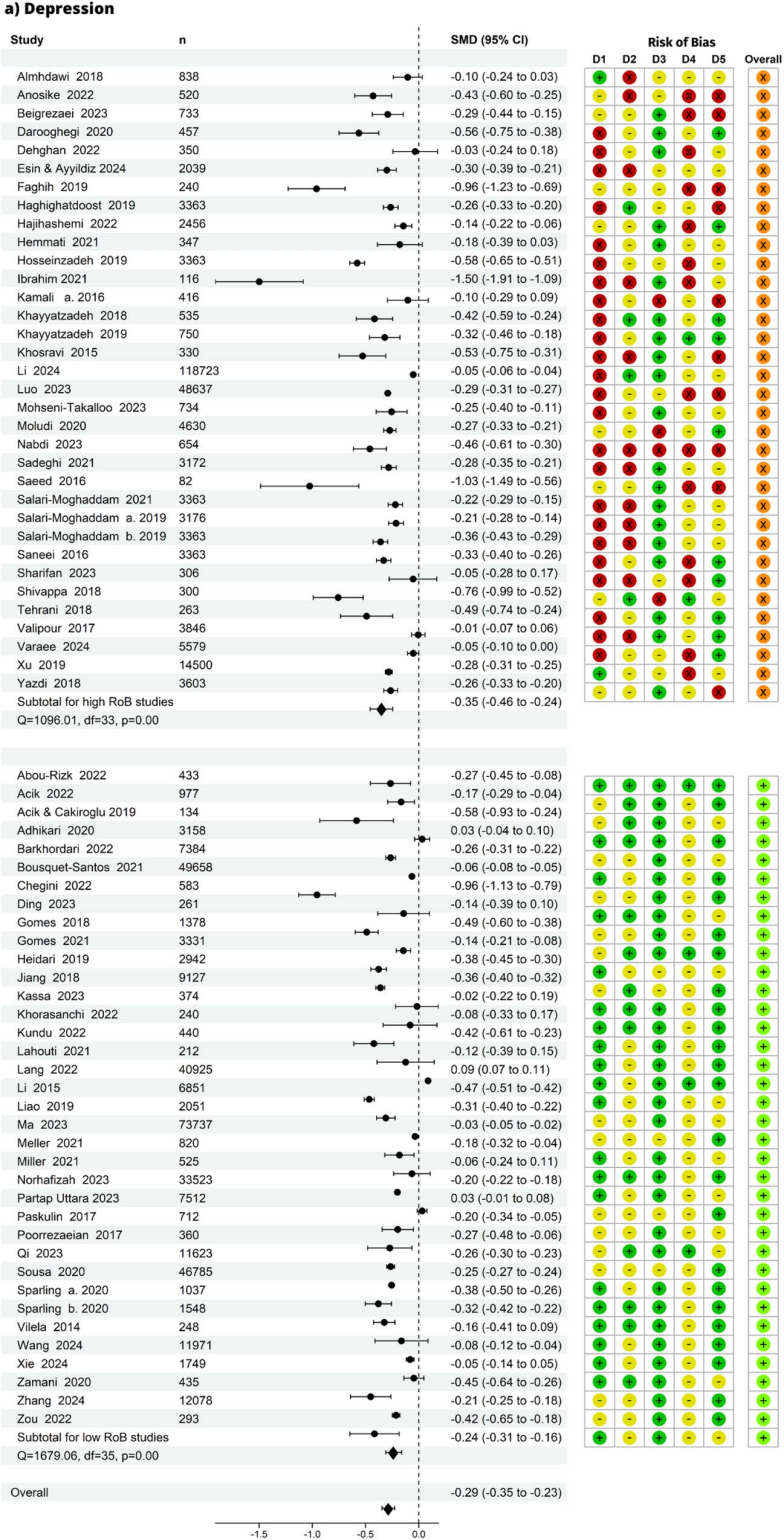

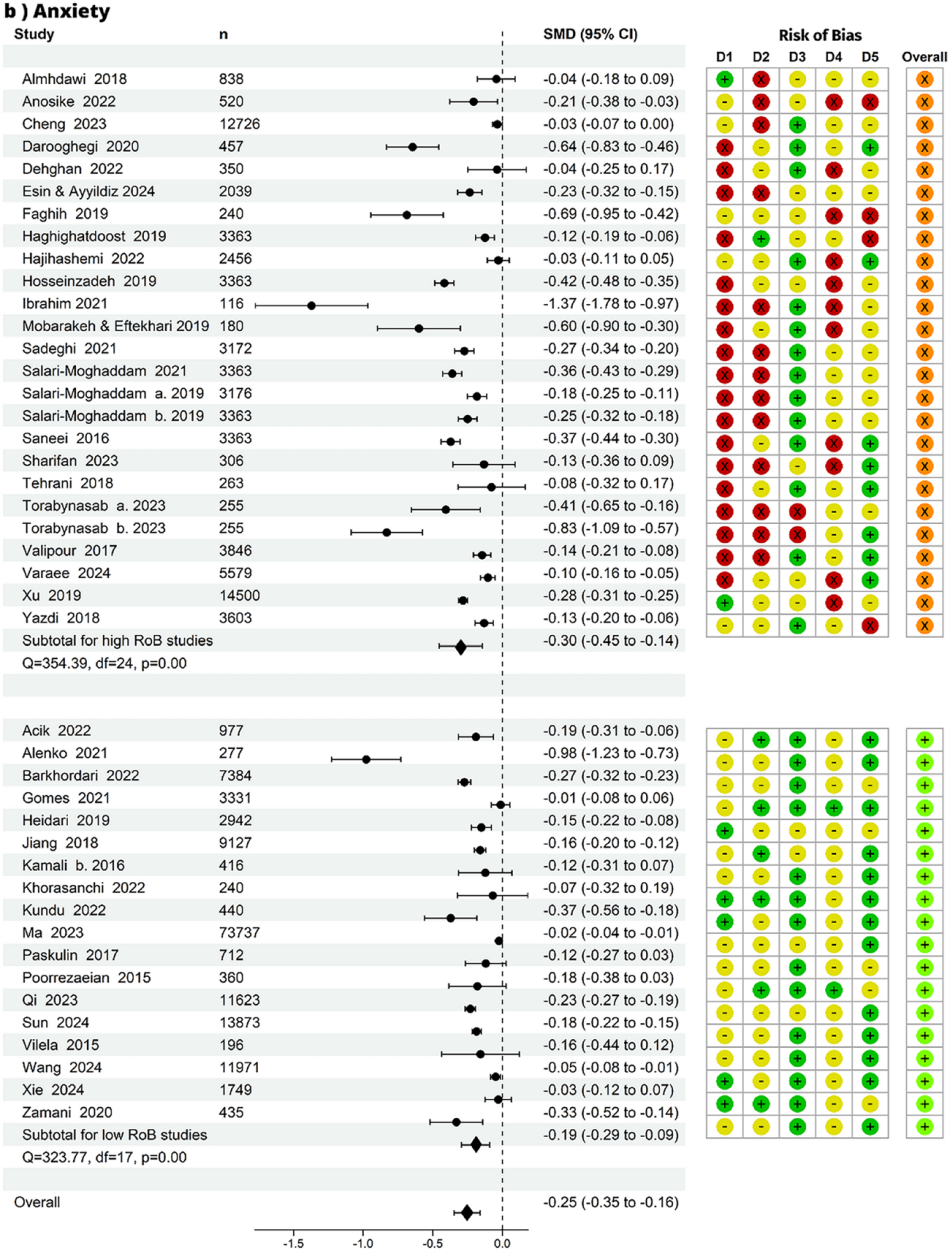

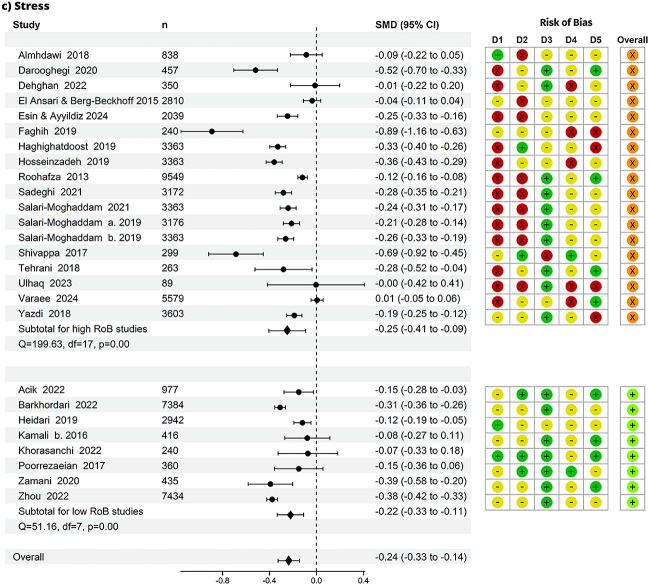
Summary effect and risk of bias (RoB). Forest plot and risk of bias per domain of all studies by mental health outcome, panel (**a**) depression, panel (**b**) anxiety, panel (**c**) stress: n=number of observations. D1 = bias due to study participation, D2 = bias due to exposure measurement, D3 = bias due to outcome measurement, D4 = bias due to confounding, D5 = bias in statistical analysis and reporting, overall = no high risk of bias in any domain. D1 to D5 RoB: green = low RoB; yellow = unclear RoB; red = high RoB. overall RoB: orange = high RoB; light green = low RoB. In addition to the overall pooled effect, each panel displays subgroup pooled effects for overall high and overall low RoB studies

All but 7 estimates were based on mental health assessments using validated instruments designed as screening tools. Two studies measured healthy diets through adherence and adequacy (G1), 22 studies measured healthy diets through adherence to patterns proven to reduce chronic disease (G2), and 15 and 19 studies, respectively, used diet diversity and diet quality indices (G3 and G4). Twenty-six studies fell into Group 5, using a posteriori methods such as factor analysis. One study used dietary measures from two groups Table 1. 

### Summary of effects

From 139 effect estimates, we pooled the effect for the association between healthy diets and depression, anxiety and stress (Fig. 3). We found that individuals with healthy diets had less depressive symptoms compared to those with unhealthy diets (SMD = -0.29, 95% CI -0.35 to -0.23), with a mean difference of 0.29 standard deviations in depression between groups. Similarly, healthy diets were associated with less anxiety (SMD = -0.25, 95% CI -0.35 to -0.16) and less stress (SMD = -0.24, 95% CI -0.33 to -0.14). The effect sizes from individual studies ranged from -1.50 to 0.09 for depression, -0.89 to 0.01 for anxiety, and -0.89 to 0.00 for stress. The prediction intervals (versus the confidence intervals) were more conservative in their estimates of the true population-level effect (Supplementary Material 1: Figure S1: A-C).

### Evidence strength

Forty-one studies that had no ‘high’ risk of bias in any domain were grouped into an overall low risk of bias group, and 42 studies with at least one high risk of bias rating in any domain were grouped into overall ‘high’. There was strong evidence of slightly weaker pooled effect estimates from studies with overall low risk of bias in each group (depression=35; anxiety=18; stress=8): depression (SMD = -0.24, CI: -0.31 to -0.16; n=266,831), anxiety (SMD = -0.19, CI: -0.29 to -0.09; n=116,248), and stress (SMD = -0.22, CI: -0.33 to -0.11; n=12,338). The rankings on ‘study attrition’ for longitudinal studies are included in Supplementary Material 1: Figure S2

Given that most studies were cross-sectional, the most likely sources of bias were study participation (domain 1) and confounding (domain 4). The ranking of potential bias from study participation was based on assessing the source of target population, method used to identify population, recruitment period, place of recruitment, inclusion and exclusion criteria, adequate study participation, and baseline characteristics. As all but 3 studies treated diet as the exposure, the risk of study participation bias stemmed from the likelihood that the relationship between diet quality and mental health is different for participants in healthy eating and unhealthy eating groups. When restricting the analysis to studies with low risk of study participation bias, the estimates remained robust: depression (SMD = -0.18, CI: -0.26 to -0.10), anxiety (SMD = -0.14, CI: -0.26 to -0.02), and stress (SMD = -0.11, CI: -0.23 to -0.01). The risk of bias from confounding was assessed based on whether important confounders (factors related both to healthy eating and mental health, such as wealth/income) were measured, defined, valid and reliably measured, had uniform method and setting across all participants, whether they followed a valid approach to deal with missing data, and if important potential confounders were accounted for in the analysis. When restricting the analysis to studies with low risk of confounding bias, we found similar results, except for stress, as there were only 2 studies in this subgroup, and with weaker evidence of these effects given the smaller sample sizes (depression SMD = -0.26, CI: -0.55 to 0.02, anxiety SMD = -0.06, CI: -1.05 to 0.92, and stress SMD = -0.25, CI: -0.73 to 0.22). Pooled estimates as well as study participation and confounding risk of bias (all low vs. high) are in Supplementary Material 1: Table S6.

### Outliers and influential studies

Two outlier studies were identified based on studentized residuals: one for depression (Ibrahim et al., 2021) and one for anxiety (Ibrahim et al., 2021). No outliers were detected for stress. Regarding influential studies, three studies were flagged for depression (Faghih et al., 2019, Ibrahim et al., 2021, and Chegini et al., 2022), two for anxiety (Alenko et al., 2021 and Ibrahim et al., 2021), and one for stress (Faghih et al., 2019). Notably, Ibrahim et al., 2021 was consistently identified as both an outlier and an influential study across multiple outcomes. When restricting the analysis to studies with low risk of bias, no outliers remained for any outcome. However, Chegini et al., 2022 remained an influential study for depression.

### Sub-group and sensitivity analyses

Findings were consistent in direction and magnitude across country income levels, study design, and dietary measurements. By country-level income classification, low-income countries had only 3 estimates for depression, 1 for anxiety and 0 for stress, while lower-middle income countries had only 4 estimates for anxiety and 2 for stress. For any country-level income group with more than 4 studies, the results were as follows: depression in lower middle-income countries, SMD -0.39 (CI: -0.71 to -0.07; *n* = 10); depression in upper middle-income countries, SMD -0.28 (CI: -0.34 to -0.22; *n* = 57); anxiety in upper-middle income countries, SMD -0.20 (CI: -0.27 to -0.13; *n* = 38), stress in upper-middle income countries SMD -0.26 (CI: -0.36 to -0.16; *n* = 24). When we restricted analyses by study design to the nine longitudinal studies, results were almost identical. By diet measure, most studies used a priori tools to measure diets associated with a reduction in nutrition-related chronic diseases, which alone produced stronger pooled effect sizes: depression SMD -0.36 (CI: -0.51 to -0.21; *n* = 18); anxiety SMD -0.25 (CI: -0.41 to -0.09; *n* = 14); and stress SMD -0.32 (CI: -0.56 to -0.09; *n* = 12) (Supplementary Material 1: Figure S3). Results were not sensitive to the choice of different effect size indices, and we report Cohen’s d and Fisher’s z transformation estimates in Supplementary Material 1: Figure S4.

**Fig. 4:**
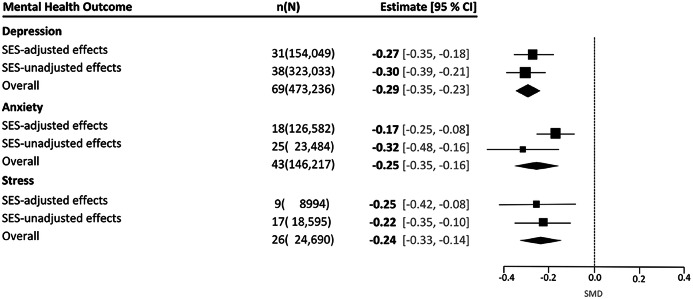
Analysis of effects on depression, anxiety and stress stratified by whether estimates included adjustment for Socio-Economic Status (SES). Adjustments included socio-economic factors measured through income, social-economic status, wealth or asset indices, family affluence scale or self-perceived economic status. Totals may not match the sum of mental health outcome populations because the analyses consider unique individuals rather than duplicated counts across categories

We further investigated the confounding effects of socio-economic factors to the extent possible in these studies. We identified that 45% of the studies (37/83) controlled for socio-economic status (SES) through income, social-economic factors, wealth, family affluence scale or self-perceived economic status. Figure 4 shows that SES-adjusted pooled effects produced similar effects sizes for depression (SMD -0.27; CI: -0.35 to -0.18; *n* = 31) and stress (SMD -0.25; CI: -0.42 to -0.08; *n* = 9). SES-adjusted anxiety effect sizes were somewhat smaller (SMD -0.17; CI: -0.25 to -0.08; *n* = 18) compared to unadjusted effect sizes (SMD -0.32; CI: -0.48 to -0.16; *n* = 25).

## Discussion

We found consistent associations between healthy diets and better mental health when pooling eligible studies from LMIC. The magnitude of pooled effects was similar for depression, anxiety and stress (-0.29, -0.25 and -0.24, respectively). We are confident that these results are not due to chance and are reasonably precise, also noting that most of the evidence was cross-sectional and none were based on counterfactual impact assessments. It is possible that the relationships we observe are due to underlying factors, such as socioeconomic deprivation, although we found evidence for similar effects when restricting the analysis to estimations adjusted for SES. Still, to our knowledge, this analysis presents the most robust estimates of these relationships in LMIC to date.

Our results signal strong evidence of modest associations, but the conversion to standardised difference effects may mask population-level significance. As such, for purposes of interpretation, we calculated that if 1% of the 1.68 billion people who cannot afford a healthy diet in LMIC moved to a ‘healthy diet’ status, these changes could be associated with 41% lower odds of depression (OR 0.59, 95% CI 0.53 to 0.66), amounting to between 0.90 and 1.27 million less cases of depression per 1% change (Supplementary Material 1: Table S7). This shows that even modest associations between healthy diets and mental health status underpin the possibility of large population-level improvements through policy and programme interventions. This is especially true as diet composition affects 100% of the global population several times a day.

Our results align with other similar meta-analyses. An umbrella review of meta-analyses included very similar measures of ‘healthy diets’ but looked only at depression, in any setting or population, but did not pool effect estimates from the included meta-analyses [52]. Overall, Gianfredi et al. found the methodological quality low to critically low, but did conclude that there was suggestive evidence linking healthy diets defined *a posteriori* with depressive symptoms or diagnosis, which was supported by a meta-analysis focusing only on *a posteriori* diet measures, and results from pooling 8 prospective studies on dietary patterns and incident depression [53, 77]. Gianfredi et al. found stronger evidence and effects for the links between higher adherence to the Mediterranean diet and lower scores on the DII and lower risk of depression. These conclusions are similar to quantitative estimates from Lassale et al., who found that the Mediterranean diet conferred a relative risk (RR) of depression of 0.67 from four longitudinal studies (95% CI: 0.55 to 0.82), and a lower DII similarly protective (RR 0.76; 95% CI: 0.63 to 0.92) from four longitudinal studies [44]. In older populations, higher DII score was also associated with incidence of depression (OR 1.33; 95% CI: 1.04 to 1.70) based on prospective studies, although they did not find associations with the Mediterranean diet or ‘healthy diet’ [78]. The Lassale et al. meta-analysis of 8 prospective cohorts and 9 cross-sectional studies on DII alone estimated that diets with higher inflammatory potential increased odds of depression by 45% (95% CI: 1.30 to 1.62) and anxiety by 66% (95% CI: 1.41 to 1.96). They also found evidence for the protective effects of a higher HEI orAlternate Healthy Eating Index (AHEI) score (RR 0.65; 95% CI: 0.50 to 0.84) from mixed longitudinal and cross-sectional studies [44]. The Gianfredi umbrella review found no evidence for vegetarian diets, also supported by another meta-analysis on this topic [54].

These synthesis studies share certain similarities with our work: they generally find that healthier diets are associated with better mental health, and even the direction and magnitude of effects are similar. Almost all note methodological limitations and heterogeneity, as we do. These studies differ in important ways from our analysis: almost all focus only on depression whereas we include anxiety and stress as well. Some mix prevention and treatment of mental health problems; we excluded treatment research since selecting participants into a study based on poor health status fundamentally confounds the relationship we were interested in testing. Some also found differences based on diet measurement, whereas our results were similar for all included measures, even if some of the evidence of effect was weak. None of the previous analyses focus on LMIC settings.

We find that most of the LMIC evidence comes from two middle-income countries: Iran and China (together 68% of included studies), even though our study includes findings from 23 LMIC. There were several studies from other places such as Brazil, Bangladesh, Ethiopia or Turkey, but these were not numerous or methodologically strong enough to draw conclusions about these different contexts. Thus, despite pooling many studies, there are still important evidence gaps to fill from low-income settings, and in LMIC other than Iran and China. Nonetheless, we show robust relationships between healthy diets and mental health symptoms.

We gain confidence and novel insights from sub-analyses, which showed similar direction and magnitude of effects despite some loss of power from smaller sub-sample sizes. For instance, when including only low risk of bias studies, the effect estimates weakened only somewhat (for depression -0.29 SMD to -0.24; for anxiety -0.25 to -0.19, and almost no change for stress). Changes could be explained by stronger study designs (which were rated lower risk of bias) that account better for the many factors at play influencing these relationships, such as controlling for socio-economic factors, a history of mental health issues or using more precise or standardised measures of diets (e.g., HEI versus a factor-derived, sample-dependent dietary pattern). We are also reassured by the similarities in estimates when only pooling effects that include adjustments for socio-economic factors (income, social-economic status, wealth, family affluence scale or self-perceived economic status) (depression: -0.27 from -0.29, anxiety: -0.17 from -0.25, and stress: -0.25 from -0.24). Especially dietary, as well as mental health, assessment are prone to large measurement bias, which generally results in attenuated effects in observational studies. Thus, it is likely that our results, as well as those of other studies, are an underestimation of the true relationship between diets and mental health.

We found consistent associations across dietary measurements, mental health screening tools and study designs. For instance, regarding dietary measurements, measuring quantitative dietary intake and calculating dietary adequacy (G1 and G4) could capture a different aspect of a ‘healthy diet’, than a qualitative count of different food groups (group 2 and group 3) or a factor analysis of dietary data in a population (G5). Reassuringly, we find similar effect estimates across dietary measurements, even when similar studies did not. Additionally, we found that certain measures were more commonly used in LMIC than others. For instance, dietary diversity, a validated measure of diet quality of women and children, is now a standard indicator in LMIC, but is less commonly used in HIC [79, 80]. In contrast, very few studies used dietary adequacy or adherence to national guidelines because national diet guidelines are both less defined and less measured in LMIC.

Although we are confident that our results are robust and precise, we also note the prediction intervals of our estimates, which are a better measure of population variance versus the sample variance. The prediction intervals from our results indicate that the true average effect of consuming a healthy diet, based on heterogeneity of the studies, could be protective or have no effect on mental health. The interpretation of the prediction intervals aligns with the heterogeneity of the studies included and may also reflect small but possibly real differences in sample populations and settings.

Our study benefits from a rigorous methodology, including a broad, thorough search of the topic to create the EGM, and screening and coding processes to identify studies following PRISMA-ScR and EGM reporting guidelines [56, 81]. We also cast a wide net for relevant studies, including multiple measures of common mental health issues, and a broad definition of ‘healthy diets’, which allowed us to compare associations across several different facets of these topics. We were able to include many studies, and carefully considered data dependencies, arriving at a three-level model accounting for overlapping study populations. This means that we neutralised the bias coming from multiple reporting of estimates produced from the same populations. We explored consistency and robustness of the evidence, finding clear associations.

We searched the literature through three databases and until January 2024, so studies indexed in non-English repositories, not in English or published after this date are not included. However, we do not believe that the results would meaningfully change based on the number of studies included from the geographies represented and countries that often publish in their dominant language are relatively well-represented in our analysis (e.g., China, Brazil). We classified country-level income status at the time of this analysis, and thus some classification may have changed (almost always to higher income status) since the time of publication or data collection.

We included studies on ‘healthy’ diets, excluding any focused solely on ‘unhealthy’ diets without a ‘healthy’ group comparator. Thus, we may have excluded some dietary measures that are in fact related to worse mental health. Practically, to pool effect estimates, we limited to similar comparators across studies. Furthermore, unhealthy diets are harder to define. ‘Unhealthy’ can mean too much consumption in general, too much of the wrong foods (e.g. foods linked with health issues such as diabetes like sugars and UPFs), too little calories (e.g. wasting and thinness), or too few micronutrients (e.g. growth faltering, reduced immunity and micronutrient deficiency). There are also increasing layers of assessment for diets that correspond to planetary rather than human health, for instance the water footprints or fertiliser demands for certain crops in certain settings, or carbon emissions for specific foods or transport processes. These relationships will also have bearing on mental health status, but this was beyond the scope of this analysis. While most of the mental health screening tools were validated, some specifically for the setting in which they were used, they may not have been used correctly or carefully, or for the outcomes for which they were designed, which could have led to measurement error or misinterpretation of mental health dimensions which they represent.

Our findings, together with other meta-analyses on related topics, indicate that healthy diets are linked to better mental health. Findings also highlight the striking need for evidence that unpacks causal mechanisms and pathways to impact, including from studies that test what changes, modifies or mediates the relationship between diets and mental health (e.g., disentangling physiological from socioeconomic effects). For instance, the role of wealth and wealth disparity, while often adjusted for within the pool of estimates, could still result in a confounding or interactive relationship between diet and mental health. We will only be able to answer these questions by design: longitudinal, prospective analyses, accounting robustly for a variety of potentially influential factors, reducing likely sources of bias, across diverse settings.

Advances in intervention research on this topic are urgently needed. To begin with, there have been proposals to standardise and improve conduct and reporting of nutritional psychiatry interventions [82]. There are promising findings that mental health can improve through dietary interventions [83–86], that counselling and integrated mental health interventions can improve nutrition outcomes [87, 88], that nutrition-sensitive interventions can improve mental health even when it is not a primary (or even secondary) outcome [89, 90], or that mental health improves through intervention components not specifically designed to improve mental health [91]. Corroborating these findings and expanding this evidence base will enable us to act on the relationships that are clear in this analysis. The potential synergies between healthy diets and better mental health could prove an important lever to address widespread burdens of poor mental health and poor diets, especially where concentrated among those most vulnerable to poverty and poor health, as well as those most at risk from increasing environmental and political instability.

From a policy perspective, building upon the interdependencies between diet and mental health is nascent, but is essential for policies and programmes focused on basic wellbeing or preventative care. For example, in Ethiopia, the Ministry of Health has demonstrated a strong commitment to integrating mental health services into national health programmes, particularly into primary health care, but also into its health extension and social protection activities for food security e.g., the National Social Protection (NSP) Policy, despite implementation challenges [92, 93]. Specifically, mental health integration has been trialled within specific branches of the Productive Safety Net Program (PSNP) by including components such as care groups and Interpersonal Psychotherapy Group (IPT-G) for Depression [94]. Mental health screening for caretakers has also been proposed as an important part of Community Management of Acute Malnutrition programmes [95, 96], and long-understood as an important step in the primary care pathway [97]. We have learned much about multisectoral programming for nutrition (e.g. on nutrition-sensitive agriculture, social protection, and school meals) [98]. Our findings could be integrated with these lessons, especially policies and programmes aiming to address multiple indicators of the Sustainable Development Goals, as well as wellbeing overall, which likely to centre in the post-2030 agenda [99, 100].

## Conclusions

We found that overall, healthy diets are associated with less depression, anxiety and stress in LMIC, with more than half of the evidence emerging from cross-sectional studies in Iran and China. Despite heterogeneity and methodological weaknesses in many studies, these results lend confidence to the robustness of associations. Our findings are foundational for further inquiry: they should spur studies from more LMIC, and settings outside of Iran and China, and they showcase the need for studies that advance our understanding of causal mechanisms and intervention research that can tell us how to activate these mechanisms through policy and programming in diverse settings.

Understanding the mechanistic and contextual factors that change the relationship between diets, and more broadly food security and nutrition, with mental health would provide a lever for integrated or co-located interventions that reduce health risks among populations that experience an unequal share of concurrent burdens and marginalisations. If we are intent on addressing global health inequity, then these relationships will be key to improving wellbeing overall.

## Supplementary Information

Below is the link to the electronic supplementary material.

Supplementary Material 1: Supplementary Tables. Supplementary Figures.

## Data Availability

Data availability and study replication: The full list of manuscripts used for data extraction are available in the Supplementary Materials. The data extraction tables, conversion methods, and reproducible code is publicly available on a GitHub repository [101] deposited on Zenodo https://doi.org/10.5281/zenodo.19614006.
