# Supplementary material for: Meta-analysis of 633,317 individuals shows associations between healthy diets and depression, anxiety and stress in 23 low- and middle-income countries
Source: BMC Glob Public Health. 2026 Jun 1;4:54. doi: 10.1186/s44263-026-00283-w (PMC13224464; doi:10.1186/s44263-026-00283-w)

SUPPLEMENTARY MATERIAL 1

# Contents

# **Supplementary Tables**

## Table S1: Example search strategy from Medline. Search for CAB Global Health and PsychInfo databases are similarly constructed

## Table S2: Checklist for Meta-analyses Of Observational Studies in Epidemiology (MOOSE)

## Table S3: Multi-step inclusion and exclusion criteria

Table S4: Quality in Prognostic Studies (QuiPS) Risk of Bias tool

Table S5: List of included studies in the meta-analysis

Table S6: Pooled estimated based on Risk of bias (low vs. high) overall and in study participation and confounding domains

Table S7: Population Attributable Fraction calculation

# **Supplementary Figures**

Figure S1A-C: Prediction intervals of the pooled effect comparing healthy diets to depression, anxiety and stress

Figure S2: Risk of bias in longitudinal studies (n=9), including study attrition domain

Figure S3: Pooled effect estimates by dietary measure group

Figure S4: Sensitivity analysis of different pooled effect measures

# Supplementary Tables

## **Supplementary Table S1:** Example search strategy from Medline. Search for CAB Global Health and PsychInfo databases are similarly constructed

| **Ovid MEDLINE(R) and In-Process & Other Non-Indexed Citations and Daily <1946 to January 31, 2024> Searched 4th January 2024**  1 ("mental health" or ((distress or stress) adj2 (psychological or psychosocial or mental)) or depression or wellbeing or "well being" or well-being or ((depressive or anxiety or anxious or affective or mood or mental) adj2 disorder*)).ti,ab,kw.  2 *depression/ or *depression, postpartum/ or *adaptation, psychological/ or *emotional adjustment/ or *survivorship/ or *mental health/ or *resilience, psychological/ or *mental disorders/ or *anxiety disorders/ or *"feeding and eating disorders"/ or *anorexia nervosa/ or *mood disorders/ or *depressive disorder/ or *adjustment disorders/ or *stress disorders, traumatic/ or *affective symptoms/dh, ec, ep, eh, et, mo, pp, pc, rh, th or *psychological trauma/ or *stress, psychological/  3 or/1-2  4 *nutrition disorders/ or *child nutrition disorders/ or *infant nutrition disorders/ or *exp malnutrition/ or *fetal nutrition disorders/ or *severe acute malnutrition/ or *kwashiorkor/ or *wasting syndrome/ or *nutritional status/  5 (nutrition* or nutrient* or micronutrient* or malnutrition or malnourish* or kwashiorkor or marasmus or undernutrition or ((iron or iodine or vitamin* or zinc) adj3 deficien*)).ti,ab,kw.  6 or/4-5  7 *dietary supplements/ or *breast feeding/ or *diet/ or *feeding behavior/ or *bottle feeding/ or *food preferences/ or *weaning/ or *food supply/ or *famine/ or *eating/ or *maternal nutritional physiological phenomena/ or *prenatal nutritional physiological phenomena/ or *nutritional requirements/ or *Infant Nutritional Physiological Phenomena/ or *Child Nutritional Physiological Phenomena/ or *infant food/ or *infant formula/  8 (diet* or ((food or eating) adj3 (pattern* or intake or shortage* or securit* or insecurit* or supplement* or access* or depriv* or belief* or consumption or cost* or purchas* or buy* or afford* or supplies or supply or availab*)) or "breast feed*" or "breast fed" or breastfeed* or breastfed or "bottle feed*" or "bottle fed" or ((infant* or child*) adj2 (feed* or food* or nutrition* or formula* or wean*))).ti,ab,kw.  9 or/7-8  10 *growth disorders/ or *fetal growth retardation/ or *fetal development/ or *fetal viability/ or *developmental disabilities/ or *pregnancy outcome/ or *pregnancy complications/px or *premature birth/ or *birth weight/ or *fetal weight/ or *thinness/ or *failure to thrive/ or *exp infant, low birth weight/ or *exp infant, premature/ or *"child of impaired parents"/ or *child development/  11 ((growth adj2 (disorder* or retard*)) or ((pregnan* or birth) adj3 outcome*) or birthweight or "birth weight" or ((premature or preterm or "pre term") adj2 (infant* or birth*)) or "failure to thrive").ti,ab,kw.  12 or/10-11  13 ((obes* or overweight or "over weight" or underweight or "under weight" or ((body or bodily) adj2 (thin or thinness))) adj5 (nutrition* or nutrient* or micronutrient* or malnutrition or malnourish* or kwashiorkor or marasmus or undernutrition or ((iron or iodine or vitamin* or zinc) adj3 deficien*))).ti,ab,kw.  14 (*obesity/dh, ep, eh, et, pc, px, rh or *obesity, maternal/dh, ep, eh, et, pc, px, rh or *obesity, morbid/dh, ep, eh, et, pc, px, rh or *pediatric obesity/dh, ep, eh, et, pc, px, rh or *overweight/dh, ep, eh, et, pc, px, rh or *thinness/dh, ep, eh, et, pc, px, rh) and (nutrition/ or nutrition disorders/ or child nutrition disorders/ or infant nutrition disorders/ or exp malnutrition/ or fetal nutrition disorders/ or severe acute malnutrition/ or kwashiorkor/ or wasting syndrome/ or nutritional status/)  15 or/13-14  16 *anthropometry/ or *apgar score/ or *"body weights and measures"/ or *body fat distribution/ or *body mass index/ or *body weight/  17 (anthropometr* or "apgar scor*" or "body measur*" or "body mass index" or BMI).ti,ab,kw.  18 or/16-17  19 6 or 9 or 12 or 15 or 18  20 ((systematic* or synthes*) adj3 (research or evaluation* or finding* or thematic* or report or descriptive or explanatory or narrative or meta* or review* or data or literature or studies or evidence or map or quantitative or study or studies or paper or impact or impacts or effect* or compar*)).ti,ab,kw.  21 ("meta regression" or "meta synth*" or "meta-synth*" or "meta analy*" or "metaanaly*" or "meta-analy*" or "metanaly*" or "metaregression" or "metaregression" or "logistic regression" or "methodologic* overview" or "pool* analys*" or "pool* data" or "quantitative* overview" or "research integration").ti,ab,kw.  22 (review adj3 (effectiveness or effects or systemat* or synth* or integrat* or map* or methodologic* or quantitative or evidence or literature)).ti,ab,kw.  23 ("meta ethnograph*" or "meta synthesis" or (synthesis and ("qualitative literature" or "qualitative research")) or "critical interpretive synthesis" or ("systematic review" and ("qualitative research" or "qualitative literature" or "qualitative stud*")) or "thematic synthesis" or "framework synthesis" or "realist review" or "realist synthesis" or "qualitative systematic review*" or "qualitative evidence synthes*" or (("quality assessment" or "critical appraisal" or "literature search*") and ("qualitative research" or "qualitative literature" or "qualitative stud*")) or (Noblit and Hare) or "meta narrative*" or "narrative synthesis").ti,ab,kw.  24 (random$ or placebo$ or single blind$ or double blind$ or triple blind$ or cohort$ or (case* adj2 control*) or ((cohort or follow up or follow-up) adj2 (control$ or series or report$ or study or studies)) or retrospective$ or ((quasi-experiment* or observ$) adj3 (study or studies or design))).ti,ab,kw.  25 meta-analysis/ or "systematic review"/ or controlled clinical trial/ or randomized controlled trial/ or random allocation/ or equivalence trial/ or pragmatic clinical trial/ or case-control studies/ or retrospective studies/ or exp cohort studies/ or controlled before-after studies/ or cross-sectional studies/ or interrupted time series analysis/ or multivariate analysis/ or qualitative research/ or risk assessment/  26 or/20-25  27 3 and 19 and 26  28 limit 27 to yr="2000 -Current" |
| --- |

## **Supplementary Table S2:** MOOSE Checklist for Meta-analyses of Observational Studies

| **Item N^o^** | **Recommendation** | **Reported** |
| --- | --- | --- |
| Reporting of background should include | | |
| 1 | Problem definition | Yes |
| 2 | Hypothesis statement | Na |
| 3 | Description of study outcome(s) | Yes |
| 4 | Type of exposure or intervention used | Yes |
| 5 | Type of study designs used | Yes |
| 6 | Study population | Yes |
| Reporting of search strategy should include | | |
| 7 | Qualifications of searchers (eg, librarians and investigators) | Yes |
| 8 | Search strategy, including time period included in the synthesis and key words | Yes |
| 9 | Effort to include all available studies, including contact with authors | Yes |
| 10 | Databases and registries searched | Yes |
| 11 | Search software used, name and version, including special features used (eg, explosion) | Yes |
| 12 | Use of hand searching (eg, reference lists of obtained articles) | Yes |
| 13 | List of citations located and those excluded, including justification | Yes |
| 14 | Method of addressing articles published in languages other than English | Na |
| 15 | Method of handling abstracts and unpublished studies | Yes |
| 16 | Description of any contact with authors | Na |
| Reporting of methods should include | | |
| 17 | Description of relevance or appropriateness of studies assembled for assessing the hypothesis to be tested | Yes |
| 18 | Rationale for the selection and coding of data (eg, sound clinical principles or convenience) | Yes |
| 19 | Documentation of how data were classified and coded (eg, multiple raters, blinding and interrater reliability) | Yes |
| 20 | Assessment of confounding (eg, comparability of cases and controls in studies where appropriate) | Yes |
| 21 | Assessment of study quality, including blinding of quality assessors, stratification or regression on possible predictors of study results | Yes |
| 22 | Assessment of heterogeneity | Yes |
| 23 | Description of statistical methods (eg, complete description of fixed or random effects models, justification of whether the chosen models account for predictors of study results, dose-response models, or cumulative meta-analysis) in sufficient detail to be replicated | Yes |
| 24 | Provision of appropriate tables and graphics | Yes |
| Reporting of results should include | | |
| 25 | Graphic summarizing individual study estimates and overall estimate | Yes |
| 26 | Table giving descriptive information for each study included | No |
| 27 | Results of sensitivity testing (eg, subgroup analysis) | Yes |
| 28 | Indication of statistical uncertainty of findings | Yes |

| **Item N^o^** | **Recommendation** | **Reported** |
| --- | --- | --- |
| Reporting of discussion should include | | |
| 29 | Quantitative assessment of bias (eg, publication bias) | Yes |
| 30 | Justification for exclusion (eg, exclusion of non-English language citations) | Yes |
| 31 | Assessment of quality of included studies | Yes |
| Reporting of conclusions should include | | |
| 32 | Consideration of alternative explanations for observed results | Yes |
| 33 | Generalization of the conclusions (ie, appropriate for the data presented and within the domain of the literature review) | Yes |
| 34 | Guidelines for future research | Yes |
| 35 | Disclosure of funding source | Yes |

*From*: Stroup DF, Berlin JA, Morton SC, et al, for the Meta-analysis Of Observational Studies in Epidemiology (MOOSE) Group. Meta-analysis of Observational Studies in Epidemiology. A Proposal for Reporting. *JAMA*. 2000;283(15):2008-2012. doi: 10.1001/jama.283.15.2008.

## **Supplementary Table S3:** Multi-step inclusion and exclusion criteria

**Step 1: Evidence Gap Map exclusion criteria relevant to diets domain**

|  | **Inclusion criteria** | **Exclusion criteria** |
| --- | --- | --- |
| Diet domain | Quantitative measures of specific food groups or dietary patterns or quality using food frequency questionnaires, dietary recall, food journals, consumption scores, indices, principal components analysis (PCA) or qualitative investigation | Dietary intake:  - Single-item foods (e.g. avocadoes, seaweed, walnuts, chocolate, therapeutic formulas, herbal remedies)  - Specialized or proprietary foods or formulas  - Probiotics and microbiome related formulas or microbiome status indicators  - Alcohol, caffeine, or stimulant foods  - Hormones, hormonal therapy  Dietary patterns:  - Studies that only describe understanding of diets and nutrition rather than actual practices.  - Perceptions, attitudes and behaviors about food or diets with no intake component (e.g. eating family dinners, weight loss, dieting)  - Emotional eating, disordered eating, night-time eating, binge eating, emotional eating scales (e.g. Power of Food index)  - Anorexia nervosa or bulimia is not considered as an FNS measure of food intake or eating patterns  - Weight loss interventions, low calorie diets, weight loss diets, change in weight or weight trajectories |
| Mental health domain | - Any measures of depression  - Studies examining two or more of the mental health measures above and/or investigate mood disorders or other affective symptoms  - Any measures of anxiety  - Any measures of perceived stress  - Any experiential measure of mental wellbeing, including health-related quality of life, mental quality of life, perceived wellbeing | - Studies in which the exposure is anti-depressants or other medication rather than the condition itself  - Studies that only refer to specific psychoses or disorders such as bipolar, dementia, Alzheimer’s, Parkinson’s, schizophrenia and addiction/smoking disorders  - Eating disorders  - Stressful event indices without accompanying experiential measures  - Post-traumatic stress or specific occupational stress (based on type of work, etc.) is not be included on its own  - Cortisol as an isolated measure of stress  - Happiness, general satisfaction (without specific mental health measures), or composite tools where the mental health components cannot be extracted. |
| Population | - General populations in any geographic location  - These may include specific age groups, specific genders or specific life-course stages such as pregnancy or older people  - Populations defined by certain equity aspects, such as low SES, income, occupations, or places of residence.  - Special populations including refugee status and prisoners will be included with a separate code | - Non-human populations  - Populations defined by specific health conditions such as heart disease, hypertension, diabetes, dementia, coeliac, anorexia, preterm birth, HIV, etc.  - Populations being treated in a clinical therapeutic setting, hospital settings  - Extremely niche populations with specific nutritional requirements such as professional athletes. |

**Step 2: Meta-analysis inclusion and exclusion criteria**

| **Inclusion criteria** | **Exclusion criteria** |
| --- | --- |
| Has any of the following dietary patterns which can be associated with healthy eating:  - Adherence to diet recommendations  - Nutrient adequacy  - Dietary Approaches to Stop Hypertension (DASH)  - Dietary Inflammatory Index  - Mediterranean diet  - Dietary diversity (MDD, IDDS)  - Any other Dietary Variety Scores  - Diet Quality Indexes (all)  - Global Diet Quality Score  - Global Dietary Index  - Healthy Eating Indexes (all)  - All comparisons between a healthy and an unhealthy diet. Unhealthy is a group with a processed, western, modern, traditional, or unhealthy diet determined for each study.  (AND)  Has an unhealthy eating comparison group | Out of scope in healthy eating domain:  (1) Only has a dietary intake of any food groups (FG) without a dietary pattern that could capture an aggregated ‘healthy diet’.  (2) Only has any of the following dietary patterns:  - Animal Source Foods (low or high)  - Breakfast  - Carb (low or high)  - Fruit/veg (low or high)  - Glycemia (load/index), insulin  - High protein (paleo, ketogenic)  - Low-fat, low calorie  - Meal frequency, skips, fasting  - Varied, diverse  - Vegetarian, vegan, omni  - Diet History Questionnaire (all)  - Vegetable Variety Score  - Outcome different from depression, anxiety or stress  - Duplicated data  - Lack of comparability  - Relevant estimate not reported |

##

##

## **Supplementary Table S4:** Quality in Prognostic Studies (QuiPS) Risk of Bias tool

Available at <https://methods.cochrane.org/sites/methods.cochrane.org.prognosis/files/uploads/QUIPS%20tool.pdf>


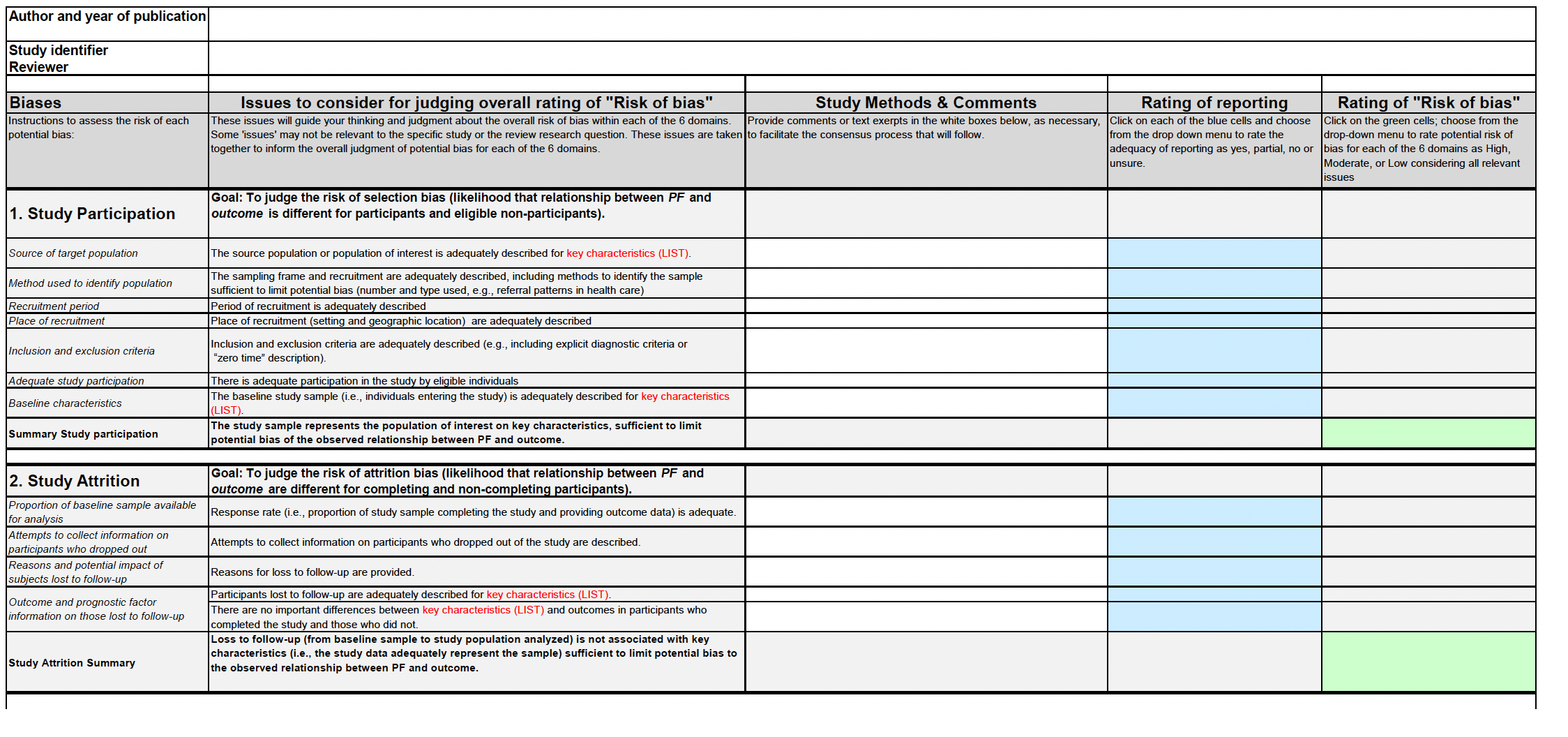


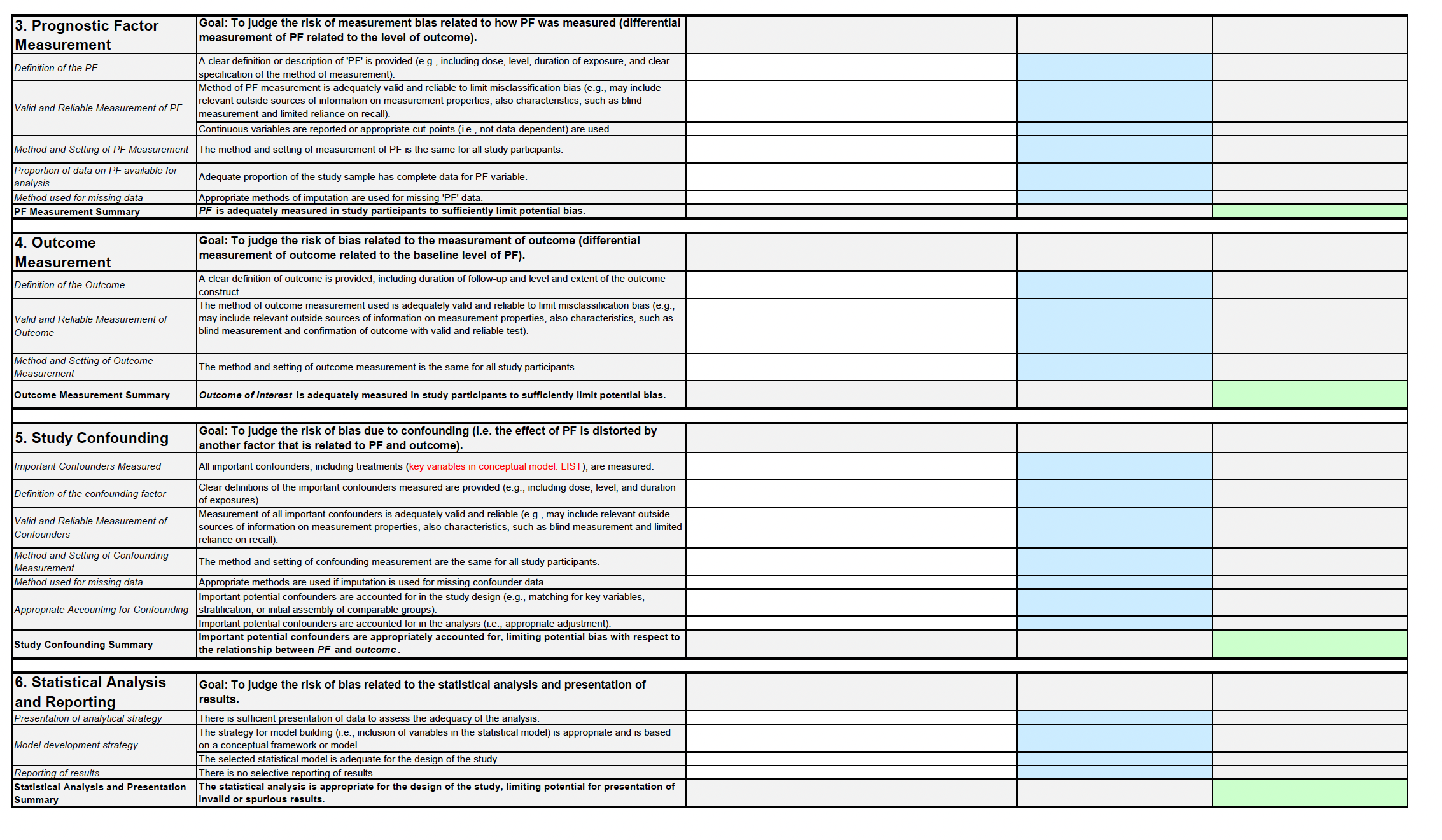


**Supplementary Table S5:** List of included studies in the meta-analysis

| **#** | **Citation** |
| --- | --- |
| 1 | Abou-Rizk J, Jeremias T, Cocuz G, Nasreddine L, Jomaa L, Hwalla N, et al. Food insecurity, low dietary diversity and poor mental health among Syrian refugee mothers living in vulnerable areas of Greater Beirut, Lebanon. Br J Nutr. 2022 Nov 14;128(9):1832–47. doi:[10.1017/S0007114521004724](https://doi.org/10.1017/S0007114521004724) |
| 2 | Açik M, Altan M, Çakiroğlu FP. A cross-sectionally analysis of two dietary quality indices and the mental health profile in female adults. Curr Psychol. 2022 Aug;41(8):5514–23. doi:[10.1007/s12144-020-01065-9](https://doi.org/10.1007/s12144-020-01065-9) |
| 3 | Açik M, Çakiroğlu FP. Evaluating the Relationship between Inflammatory Load of a Diet and Depression in Young Adults. Ecology of Food and Nutrition. 2019 Jul 4;58(4):366–78. doi:[10.1080/03670244.2019.1602043](https://doi.org/10.1080/03670244.2019.1602043) |
| 4 | Adhikari RP, Williamson R, Sparling TM, Ferguson E, Cunningham K. Parental depression and nutrition: findings from a cross-sectional household survey in Nepal. Public Health Nutr. 2020 Nov;23(16):2983–93. doi:[10.1017/S1368980020000968](https://doi.org/10.1017/S1368980020000968) |
| 5 | Alenko A, Agenagnew L, Beressa G, Tesfaye Y, Woldesenbet YM, Girma S. COVID-19-Related Anxiety and Its Association with Dietary Diversity Score Among Health Care Professionals in Ethiopia: A Web-Based Survey. JMDH. 2021 Apr;Volume 14:987–96. doi:[10.2147/JMDH.S305164](https://doi.org/10.2147/JMDH.S305164) |
| 6 | Almhdawi KA, Kanaan SF, Khader Y, Al-Hourani Z, Almomani F, Nazzal M. Study-related mental health symptoms and their correlates among allied health professions students. WOR. 2018 Dec 5;61(3):391–401. doi:[10.3233/WOR-182815](https://doi.org/10.3233/WOR-182815) |
| 7 | Anosike C, Anene-Okeke CG, Ayogu EE, Oshigbo MC. Prevalence of depression and anxiety, and attitudes toward seeking help among first-year pharmacy, medical, and nursing students at a Nigerian university. Currents in Pharmacy Teaching and Learning. 2022 Jun;14(6):720–8. doi:[10.1016/j.cptl.2022.06.002](https://doi.org/10.1016/j.cptl.2022.06.002) |
| 8 | Barkhordari R, Namayandeh M, Mirzaei M, Sohouli MH, Hosseinzadeh M. The relation between MIND diet with psychological disorders and psychological stress among Iranian adults. BMC Psychiatry. 2022 Dec;22(1):496. doi:[10.1186/s12888-022-04128-2](https://doi.org/10.1186/s12888-022-04128-2) |
| 9 | Beigrezaei S, Darabi Z, Davies IG, Mazidi M, Ghayour-Mobarhan M, Khayyatzadeh SS. Higher global diet quality score is related to lower prevalence of depression and poor quality of life among adolescent girls. BMC Psychiatry. 2023 Nov 28;23(1):886. doi:[10.1186/s12888-023-05313-7](https://doi.org/10.1186/s12888-023-05313-7) |
| 10 | Bousquet-Santos K, Chen R, Kubzansky LD. A sad heart: Depression and favorable cardiovascular health in Brazil. Preventive Medicine. 2021 Jan;142:106378. doi:[10.1016/j.ypmed.2020.106378](https://doi.org/10.1016/j.ypmed.2020.106378) |
| 11 | Chegini M, Shirani P, Omidvar N, Eini-Zinab H, Pour-Ebrahim F, Rezazadeh A. Relationship between diet quality and depression among Iranian older adults in Tehran. BMC Geriatr. 2022 Aug 26;22(1):708. doi:[10.1186/s12877-022-03380-1](https://doi.org/10.1186/s12877-022-03380-1) |
| 12 | Cheng T, Fu M, Zhang B, Luo L, Guo J. Do living arrangements and health behaviors associate with anxiety symptoms among Chinese older people? Differences between urban and rural areas. Psychology, Health & Medicine. 2023 Nov 26;28(10):3117–30. doi:[10.1080/13548506.2023.2224582](https://doi.org/10.1080/13548506.2023.2224582) |
| 13 | Darooghegi Mofrad M, Siassi F, Guilani B, Bellissimo N, Suitor K, Azadbakht L. The association of food quality index with mental health in women: a cross-sectional study. BMC Res Notes. 2020 Dec;13(1):557. doi:[10.1186/s13104-020-05401-x](https://doi.org/10.1186/s13104-020-05401-x) |
| 14 | Dehghan P, Nejati M, Vahid F, Almasi-Hashiani A, Saleh-Ghadimi S, Parsi R, et al. The association between dietary inflammatory index, dietary antioxidant index, and mental health in adolescent girls: an analytical study. BMC Public Health. 2022 Aug 9;22(1):1513. doi:[10.1186/s12889-022-13879-2](https://doi.org/10.1186/s12889-022-13879-2) |
| 15 | Ding Y, Li G, Shi X, Wang M, Peng Y, Deng H, et al. Correlation of lifestyle behaviors during pregnancy with postpartum depression status of puerpera in the rural areas of South China. Front Public Health. 2023 Dec 18;11:1304226. doi:[10.3389/fpubh.2023.1304226](https://doi.org/10.3389/fpubh.2023.1304226) |
| 16 | El Ansari W, Berg-Beckhoff G. Nutritional Correlates of Perceived Stress among University Students in Egypt. IJERPH. 2015 Nov 6;12(11):14164–76. doi:[10.3390/ijerph121114164](https://doi.org/10.3390/ijerph121114164) |
| 17 | Esin K, Ayyıldız F. Food insecurity, Mediterranean diet adherence, and psychosocial health among university students in Türkiye. International Journal of Environmental Health Research. 2024 Jan 2;34(1):649–59. doi:[10.1080/09603123.2023.2300405](https://doi.org/10.1080/09603123.2023.2300405) |
| 18 | Faghih S, Babajafari S, Mirzaei A, Akhlaghi M. Adherence to the dietary approaches to stop hypertension (DASH) dietary pattern and mental health in Iranian university students. Eur J Nutr. 2020 Apr;59(3):1001–11. doi:[10.1007/s00394-019-01961-2](https://doi.org/10.1007/s00394-019-01961-2) |
| 19 | Gomes AP, Gonçalves H, Dos Santos Vaz J, Kieling C, Rohde LA, Oliveira IO, et al. Do inflammation and adiposity mediate the association of diet quality with depression and anxiety in young adults? Clinical Nutrition. 2021 May;40(5):2800–8. doi:[10.1016/j.clnu.2021.03.028](https://doi.org/10.1016/j.clnu.2021.03.028) |
| 20 | Gomes AP, Oliveira Bierhals I, Gonçalves Soares AL, Hellwig N, Tomasi E, Formoso Assunção MC, et al. Interrelationship between Diet Quality and Depressive Symptoms in Elderly. The Journal of nutrition, health and aging. 2018 Mar;22(3):387–92. doi:[10.1007/s12603-017-0963-7](https://doi.org/10.1007/s12603-017-0963-7) |
| 21 | Haghighatdoost F, Feizi A, Esmaillzadeh A, Feinle-Bisset C, Keshteli AH, Afshar H, et al. Association between the dietary inflammatory index and common mental health disorders profile scores. Clinical Nutrition. 2019 Aug;38(4):1643–50. doi:[10.1016/j.clnu.2018.08.016](https://doi.org/10.1016/j.clnu.2018.08.016) |
| 22 | Hajihashemi P, Haghighatdoost F, Mohammadifard N, Maghroun M, Sajjadi F, Najafi F, et al. The association of dietary macronutrient quality indices with depression and anxiety symptoms and quality of life in Iranian adults: The LipoKAP study. Journal of Affective Disorders. 2022 Nov;317:409–16. doi:[10.1016/j.jad.2022.08.046](https://doi.org/10.1016/j.jad.2022.08.046) |
| 23 | Heidari Z, Feizi A, Roohafza H, Rabiei K, Sarrafzadegan N. Are dietary patterns differently associated with differentiated levels of mental health problems? Results from a large cross-sectional study among Iranian manufacturing employees. BMJ Open. 2019 Jan;9(1):e020083. doi:[10.1136/bmjopen-2017-020083](https://doi.org/10.1136/bmjopen-2017-020083) |
| 24 | Hemmati A, Ghoreishy SM, Karami K, Imani H, Farsani GM, Mousavi SE, et al. The association between dietary patterns and depression in adolescents: A cross-sectional study. Clinical Nutrition ESPEN. 2021 Dec;46:271–5. doi:[10.1016/j.clnesp.2021.09.743](https://doi.org/10.1016/j.clnesp.2021.09.743) |
| 25 | Hosseinzadeh M, Vafa MR, Esmaillzadeh A, Feizi A, Majdzadeh R, Afshar H, et al. Psychological disorders and dietary patterns by reduced-rank regression. Eur J Clin Nutr. 2019 Mar;73(3):408–15. doi:[10.1038/s41430-019-0399-8](https://doi.org/10.1038/s41430-019-0399-8) |
| 26 | Ibrahim MO. Association between major socio-demographic and psychosocial factors with the dietary pattern among elderly people in Amman, Jordan. Nutr Clín Diet Hosp. 2021 May 1;41(2). doi:[10.12873/412mohammed](https://doi.org/10.12873/412mohammed) |
| 27 | Jiang W, Mo M, Li M, Wang S, Muyiduli X, Shao B, et al. The relationship of dietary diversity score with depression and anxiety among prenatal and post‐partum women. J of Obstet and Gynaecol. 2018 Oct;44(10):1929–36. doi:[10.1111/jog.13728](https://doi.org/10.1111/jog.13728) |
| 28 | Kamali M, Dastsouz F, Sadeghi F, Amanat S, Akhlaghi M. Associations between Western and Mediterranean-type dietary patterns and anxiety and stress. Acta Alimentaria. 2016 Sep;45(3):398–405. doi:[10.1556/066.2016.45.3.11](https://doi.org/10.1556/066.2016.45.3.11) |
| 29 | Kassa G, Batchelder A, Gross D. Prevalence and determinants of postpartum depression among adolescent and adult mothers in Northwest Ethiopia. Research in Nursing & Health. 2024 Apr;47(2):125–40. doi:[10.1002/nur.22362](https://doi.org/10.1002/nur.22362) |
| 30 | Khayyatzadeh SS, Mehramiz M, Mirmousavi SJ, Mazidi M, Ziaee A, Kazemi-Bajestani SMR, et al. Adherence to a Dash-style diet in relation to depression and aggression in adolescent girls. Psychiatry Research. 2018 Jan;259:104–9. doi:[10.1016/j.psychres.2017.09.075](https://doi.org/10.1016/j.psychres.2017.09.075) |
| 31 | Khayyatzadeh SS, Shafiee M, Far PE, Ziaee SS, Bagherniya M, Ebrahimi S, et al. Adherence to a healthy dietary pattern is associated with less severe depressive symptoms among adolescent girls. Psychiatry Research. 2019 Feb;272:467–73. doi:[10.1016/j.psychres.2018.12.164](https://doi.org/10.1016/j.psychres.2018.12.164) |
| 32 | Khorasanchi Z, Ahmadihoseini A, Hajhoseini O, Zare-Feyzabadi R, Haghighi M, Heidari M, et al. Adherence to dietary approaches to stop hypertension (DASH) diet in relation to psychological function in recovered COVID-19 patients: a case–control study. BMC Nutr. 2022 Nov 11;8(1):130. doi:[10.1186/s40795-022-00633-5](https://doi.org/10.1186/s40795-022-00633-5) |
| 33 | Khosravi M, Sotoudeh G, Majdzadeh R, Nejati S, Darabi S, Raisi F, et al. Healthy and Unhealthy Dietary Patterns Are Related to Depression: A Case-Control Study. Psychiatry Investig. 2015;12(4):434. doi:[10.4306/pi.2015.12.4.434](https://doi.org/10.4306/pi.2015.12.4.434) |
| 34 | Kundu S, Rejwana N, Al Banna MdH, Kawuki J, Ghosh S, Alshahrani NZ, et al. Linking Depressive and Anxiety Symptoms with Diet Quality of University Students: A Cross-Sectional Study during the COVID-19 Pandemic in India. Healthcare. 2022 Sep 23;10(10):1848. doi:[10.3390/healthcare10101848](https://doi.org/10.3390/healthcare10101848) |
| 35 | Lahouti M, Zavoshy R, Noroozi M, Rostami R, Gholamalizadeh M, Rashidkhani B, et al. Dietary patterns and depressive symptoms among Iranian women. J Health Psychol. 2021 Oct;26(12):2278–89. doi:[10.1177/1359105320909888](https://doi.org/10.1177/1359105320909888) |
| 36 | Lang X, Liu Z, Islam S, Han G, Rangarajan S, Tse LA, et al. Interaction of Depression and Unhealthy Diets on the Risk of Cardiovascular Diseases and All-Cause Mortality in the Chinese Population: A PURE Cohort Substudy. Nutrients. 2022 Dec 5;14(23):5172. doi:[10.3390/nu14235172](https://doi.org/10.3390/nu14235172) |
| 37 | Li L, Yang P, Duan Y, Xie J, Liu M, Zhou Y, et al. Association between dietary diversity, sedentary time outside of work and depressive symptoms among knowledge workers: a multi-center cross-sectional study. BMC Public Health. 2024 Jan 2;24(1):53. doi:[10.1186/s12889-023-17567-7](https://doi.org/10.1186/s12889-023-17567-7) |
| 38 | Li Z, Yang X, Wang A, Qiu J, Wang W, Song Q, et al. Association between Ideal Cardiovascular Health Metrics and Depression in Chinese Population: A Cross-sectional Study. Sci Rep. 2015 Jul 15;5(1):11564. doi:[10.1038/srep11564](https://doi.org/10.1038/srep11564) |
| 39 | Liao K, Gu Y, Liu M, Fu J, Wang X, Yang G, et al. Association of dietary patterns with depressive symptoms in Chinese postmenopausal women. Br J Nutr. 2019 Nov 28;122(10):1168–74. doi:[10.1017/S0007114519001867](https://doi.org/10.1017/S0007114519001867) |
| 40 | Luo Y, Yang P, Wan Z, Kang Y, Dong X, Li Y, et al. Dietary diversity, physical activity and depressive symptoms among middle-aged women: A cross-sectional study of 48,637 women in China. Journal of Affective Disorders. 2023 Jan;321:147–52. doi:[10.1016/j.jad.2022.10.038](https://doi.org/10.1016/j.jad.2022.10.038) |
| 41 | Ma S, Zhu J, Xie S, Chen R, Li X, Wei W. Suboptimal dietary quality is associated with mental symptoms among adults aged 40 years and over in China: A population-based cross-sectional study. Journal of Affective Disorders. 2023 Nov;340:802–11. doi:[10.1016/j.jad.2023.08.071](https://doi.org/10.1016/j.jad.2023.08.071) |
| 42 | Meller FDO, Manosso LM, Schäfer AA. The influence of diet quality on depression among adults and elderly: A population-based study. Journal of Affective Disorders. 2021 Mar;282:1076–81. doi:[10.1016/j.jad.2020.12.155](https://doi.org/10.1016/j.jad.2020.12.155) |
| 43 | Miller LC, Neupane S, Sparling TM, Shrestha M, Joshi N, Lohani M, et al. Maternal depression is associated with less dietary diversity among rural Nepali children. Maternal & Child Nutrition. 2021 Oct;17(4):e13221. doi:[10.1111/mcn.13221](https://doi.org/10.1111/mcn.13221) |
| 44 | Mohseni-Takalloo S, Salehi-Abargouei A, Ferns GA, Ghayour-Mobarhan M, Khayyatzadeh SS. Adherence to the Healthy Eating Index-2015 and Its Association with Depression Score in A Sample of Iranian Adolescent Girls. JNFS. 2023 Jul 30. doi:[10.18502/jnfs.v8i3.13292](https://doi.org/10.18502/jnfs.v8i3.13292) |
| 45 | Moludi J, Moradinazar M, Hamzeh B, Najafi F, Pasdar Y. Depression Relationship with Dietary Patterns and Dietary Inflammatory Index in Women: Result from Ravansar Cohort Study. NDT. 2020 Jun;Volume 16:1595–603. doi:[10.2147/NDT.S255912](https://doi.org/10.2147/NDT.S255912) |
| 46 | Nabdi S, Boujraf S, Benzagmout M. The influence of physical activity, social relationships, and diet intake on depression: a case-series study. Annals of Medicine & Surgery. 2023 May;85(5):1395–402. doi:[10.1097/MS9.0000000000000406](https://doi.org/10.1097/MS9.0000000000000406) |
| 47 | Partap U, Nyundo A, Manu A, Regan M, Ismail A, Chukwu A, et al. Depressive symptoms among adolescents in six sub-Saharan African countries: A pooled analysis of associated factors. Preventive Medicine Reports. 2023 Dec;36:102499. doi:[10.1016/j.pmedr.2023.102499](https://doi.org/10.1016/j.pmedr.2023.102499) |
| 48 | Paskulin JTA, Drehmer M, Olinto MT, Hoffmann JF, Pinheiro AP, Schmidt MI, et al. Association between dietary patterns and mental disorders in pregnant women in Southern Brazil. Rev Bras Psiquiatr. 2017 Mar 23;39(3):208–15. doi:[10.1590/1516-4446-2016-2016](https://doi.org/10.1590/1516-4446-2016-2016) |
| 49 | Poorrezaeian M, Siassi F, Milajerdi A, Qorbani M, Karimi J, Sohrabi-Kabi R, et al. Depression is related to dietary diversity score in women: a cross-sectional study from a developing country. Ann Gen Psychiatry. 2017 Dec;16(1):39. doi:[10.1186/s12991-017-0162-2](https://doi.org/10.1186/s12991-017-0162-2) |
| 50 | Poorrezaeian M, Siassi F, Qorbani M, Karimi J, Koohdani F, Asayesh H, et al. Association of dietary diversity score with anxiety in women. Psychiatry Research. 2015 Dec;230(2):622–7. doi:[10.1016/j.psychres.2015.10.016](https://doi.org/10.1016/j.psychres.2015.10.016) |
| 51 | Qi R, Sheng B, Zhou L, Chen Y, Sun L, Zhang X. Association of Plant-Based Diet Indices and Abdominal Obesity with Mental Disorders among Older Chinese Adults. Nutrients. 2023 Jun 12;15(12):2721. doi:[10.3390/nu15122721](https://doi.org/10.3390/nu15122721) |
| 52 | Roohafza H, Sarrafzadegan N, Sadeghi M, Sajjadi F, Khosravi-Boroujeni H. The Association between Stress Levels and Food Consumption among Iranian Population. Archives of Iranian Medicine. 2013;16(3):145–8. |
| 53 | Sadeghi O, Keshteli AH, Afshar H, Esmaillzadeh A, Adibi P. Adherence to Mediterranean dietary pattern is inversely associated with depression, anxiety and psychological distress. Nutritional Neuroscience. 2021 Apr 3;24(4):248–59. doi:[10.1080/1028415X.2019.1620425](https://doi.org/10.1080/1028415X.2019.1620425) |
| 54 | Saeed A, Raana T, Saeed AM, Humayun A. Effect of antenatal depression on maternal dietary intake and neonatal outcome: a prospective cohort. Nutr J. 2015 Dec;15(1):64. doi:[10.1186/s12937-016-0184-7](https://doi.org/10.1186/s12937-016-0184-7) |
| 55 | Sahril N, Adnan MAA, Khalil MKN, Chan YM, Yoga Ratnam KK, Lai WK, et al. Association of dietary behaviour and depression among adolescents in Malaysia: a cross-sectional study. J Health Popul Nutr. 2023 Nov 28;42(1):133. doi:[10.1186/s41043-023-00480-5](https://doi.org/10.1186/s41043-023-00480-5) |
| 56 | Salari-Moghaddam A, Keshteli AH, Afshar H, Esmaillzadeh A, Adibi P. Association between dietary inflammatory index and psychological profile in adults. Clinical Nutrition. 2019 Oct;38(5):2360–8. doi:[10.1016/j.clnu.2018.10.015](https://doi.org/10.1016/j.clnu.2018.10.015) |
| 57 | Salari-Moghaddam A, Keshteli AH, Afshar H, Esmaillzadeh A, Adibi P. Empirically derived food-based dietary inflammatory index is associated with increased risk of psychological disorders in women. Nutritional Neuroscience. 2021 Apr 3;24(4):260–8. doi:[10.1080/1028415X.2019.1621044](https://doi.org/10.1080/1028415X.2019.1621044) |
| 58 | Salari-Moghaddam A, Keshteli AH, Mousavi SM, Afshar H, Esmaillzadeh A, Adibi P. Adherence to the MIND diet and prevalence of psychological disorders in adults. Journal of Affective Disorders. 2019 Sep;256:96–102. doi:[10.1016/j.jad.2019.05.056](https://doi.org/10.1016/j.jad.2019.05.056) |
| 59 | Salehi Mobarakeh M, Eftekhari MH. The Association between Diet Quality and Anxiety among Young Couples in Shiraz: A Cross-sectional Study. JNFS. 2019 Jul 31. doi:[10.18502/jnfs.v4i3.1309](https://doi.org/10.18502/jnfs.v4i3.1309) |
| 60 | Saneei P, Hajishafiee M, Keshteli AH, Afshar H, Esmaillzadeh A, Adibi P. Adherence to Alternative Healthy Eating Index in relation to depression and anxiety in Iranian adults. Br J Nutr. 2016 Jul 28;116(2):335–42. doi:[10.1017/S0007114516001926](https://doi.org/10.1017/S0007114516001926) |
| 61 | Sharifan P, Darroudi S, Rafiee M, Toussi MSE, Sedgh Doust FN, Taghizadeh N, et al. Association of dietary and blood inflammatory indicators with depression, anxiety, and stress in adults with vitamin D deficiency. Int J Geriat Psychiatry. 2023 Aug;38(8):e5972. doi:[10.1002/gps.5972](https://doi.org/10.1002/gps.5972) |
| 62 | Shivappa N, Hebert JR, Rashidkhani B. Association between Inflammatory Potential of Diet and Stress Levels in Adolescent Women in Iran. Archives of Iranian Medicine. 2017;20(2):108–12. |
| 63 | Shivappa N, Hebert JR, Neshatbini Tehrani A, Bayzai B, Naja F, Rashidkhani B. A Pro-Inflammatory Diet Is Associated With an Increased Odds of Depression Symptoms Among Iranian Female Adolescents: A Cross-Sectional Study. Front Psychiatry. 2018 Aug 29;9:400. doi:[10.3389/fpsyt.2018.00400](https://doi.org/10.3389/fpsyt.2018.00400) |
| 64 | Sousa KTD, Marques ES, Levy RB, Azeredo CM. Food consumption and depression among Brazilian adults: results from the Brazilian National Health Survey, 2013. Cad Saúde Pública. 2020;36(1):e00245818. doi:[10.1590/0102-311x00245818](https://doi.org/10.1590/0102-311x00245818) |
| 65 | Sparling TM, Waid JL, Wendt AS, Gabrysch S. Depression among women of reproductive age in rural Bangladesh is linked to food security, diets and nutrition. Public Health Nutr. 2020 Mar;23(4):660–73. doi:[10.1017/S1368980019003495](https://doi.org/10.1017/S1368980019003495) |
| 66 | Sun Q, Wang H, Zhang H, Zhang F. Association between the healthy eating index and anxiety among Chinese elderly: A population-based cross-sectional study. Preventive Medicine Reports. 2024 Jan;37:102576. doi:[10.1016/j.pmedr.2023.102576](https://doi.org/10.1016/j.pmedr.2023.102576) |
| 67 | Tehrani AN, Salehpour A, Beyzai B, Farhadnejad H, Moloodi R, Hekmatdoost A, et al. Adherence to Mediterranean dietary pattern and depression, anxiety and stress among high-school female adolescents. MNM. 2018 Mar 30;11(1):73–83. doi:[10.3233/MNM-17192](https://doi.org/10.3233/MNM-17192) |
| 68 | Torabynasab K, Shahinfar H, Effatpanah M, Jazayeri S, Azadbakht L, Abolghasemi J, et al. Association between empirical dietary inflammatory index, odds, and severity of anxiety disorders: A case–control study. Food Science & Nutrition. 2023 Oct;11(10):6349–59. doi:[10.1002/fsn3.3573](https://doi.org/10.1002/fsn3.3573) |
| 69 | Torabynasab K, Shahinfar H, Jazayeri S, Effatpanah M, Azadbakht L, Abolghasemi J. Adherence to the MIND diet is inversely associated with odds and severity of anxiety disorders: a case–control study. BMC Psychiatry. 2023 May 10;23(1):330. doi:[10.1186/s12888-023-04776-y](https://doi.org/10.1186/s12888-023-04776-y) |
| 70 | Ulhaq ND, Amalia DP, Rafa KD, Rizkiya I, Astuti YD, Febriyanti F, et al. Correlation between Stress and Eating Behaviour in College Students: A Longitudinal Study. HAYATI J Biosci. 2022 Aug 29;30(1):88–94. doi:[10.4308/hjb.30.1.88-94](https://doi.org/10.4308/hjb.30.1.88-94) |
| 71 | Valipour G, Esmaillzadeh A, Azadbakht L, Afshar H, Hassanzadeh A, Adibi P. Adherence to the DASH diet in relation to psychological profile of Iranian adults. Eur J Nutr. 2017 Feb;56(1):309–20. doi:[10.1007/s00394-015-1081-0](https://doi.org/10.1007/s00394-015-1081-0) |
| 72 | Varaee H, Mirzaei M, Salehi-Abargouei A, Ahmadi N, Hosseinzadeh M. Evaluation of lifestyle and dietary inflammatory score and their relationship with the odds of depression, stress, and anxiety in adults living in Yazd, Iran; based on YaHS and TAMYZ cohort study. Journal of Affective Disorders. 2024 Feb;347:486–91. doi:[10.1016/j.jad.2023.11.069](https://doi.org/10.1016/j.jad.2023.11.069) |
| 73 | Vilela AAF, Pinto TDJP, Rebelo F, Benaim C, Lepsch J, Dias-Silva CH, et al. Association of Prepregnancy Dietary Patterns and Anxiety Symptoms from Midpregnancy to Early Postpartum in a Prospective Cohort of Brazilian Women. Journal of the Academy of Nutrition and Dietetics. 2015 Oct;115(10):1626–35. doi:[10.1016/j.jand.2015.01.007](https://doi.org/10.1016/j.jand.2015.01.007) |
| 74 | Vilela AAF, Farias DR, Eshriqui I, Vaz JDS, Franco-Sena AB, Castro MBT, et al. Prepregnancy Healthy Dietary Pattern Is Inversely Associated with Depressive Symptoms among Pregnant Brazilian Women. The Journal of Nutrition. 2014 Oct;144(10):1612–8. doi:[10.3945/jn.114.190488](https://doi.org/10.3945/jn.114.190488) |
| 75 | Wang X, Yin Z, Yang Y, Fu X, Guo C, Pu K, et al. Association of plant-based dietary patterns with depression and anxiety symptoms in Chinese older adults: A nationwide study. Journal of Affective Disorders. 2024 Apr;350:838–46. doi:[10.1016/j.jad.2024.01.159](https://doi.org/10.1016/j.jad.2024.01.159) |
| 76 | Xie X, Li Y, Zhang Y, Lin X, Huang M, Fu H, et al. Associations of diet quality and daily free sugar intake with depressive and anxiety symptoms among Chinese adolescents. Journal of Affective Disorders. 2024 Apr;350:550–8. doi:[10.1016/j.jad.2024.01.101](https://doi.org/10.1016/j.jad.2024.01.101) |
| 77 | Xu H, Sun Y, Wan Y, Zhang S, Xu H, Yang R, et al. Eating pattern and psychological symptoms: A cross-sectional study based on a national large sample of Chinese adolescents. Journal of Affective Disorders. 2019 Feb;244:155–63. doi:[10.1016/j.jad.2018.10.090](https://doi.org/10.1016/j.jad.2018.10.090) |
| 78 | Yazdi M, Roohafza H, Feizi A, Rabiei K, Sarafzadegan N. The influence of dietary patterns and stressful life events on psychological problems in a large sample of Iranian industrial employees: Structural equations modeling approach. Journal of Affective Disorders. 2018 Aug;236:140–8. doi:[10.1016/j.jad.2018.04.120](https://doi.org/10.1016/j.jad.2018.04.120) |
| 79 | Zamani B, Daneshzad E, Siassi F, Guilani B, Bellissimo N, Azadbakht L. Association of plant-based dietary patterns with psychological profile and obesity in Iranian women. Clinical Nutrition. 2020 Jun;39(6):1799–808. doi:[10.1016/j.clnu.2019.07.019](https://doi.org/10.1016/j.clnu.2019.07.019) |
| 80 | Zhang X, Zhou W, Wang H, Bai Y, Zhang F, Lu W. Association between healthy eating and depression symptoms among Chinese older adults: A cross-sectional study based on the Chinese Longitudinal Healthy Longevity survey. Preventive Medicine Reports. 2024 Feb;38:102616. doi:[10.1016/j.pmedr.2024.102616](https://doi.org/10.1016/j.pmedr.2024.102616) |
| 81 | Zhou J, Wang H, Zou Z. Inverse Association between Dietary Diversity Score Calculated from the Diet Quality Questionnaire and Psychological Stress in Chinese Adults: A Prospective Study from China Health and Nutrition Survey. Nutrients. 2022 Aug 12;14(16):3297. doi:[10.3390/nu14163297](https://doi.org/10.3390/nu14163297) |
| 82 | Zou H, Sun M, Liu Y, Xi Y, Xiang C, Yong C, et al. Relationship between Dietary Inflammatory Index and Postpartum Depression in Exclusively Breastfeeding Women. Nutrients. 2022 Nov 25;14(23):5006. doi:[10.3390/nu14235006](https://doi.org/10.3390/nu14235006) |

**Supplementary Table S6:** Pooled estimated based on Risk of bias (RoB) grouped by low and high in overall RoB, in study participation RoB and confounding RoB

| RoB domain | RoB Level | Depression  SMD and 95% CI | Anxiety  SMD and 95% CI | Stress  SMD and 95% CI |
| --- | --- | --- | --- | --- |
| Overall RoB | Low | -0.23 (-0.31, -0.16) | -0.19 (-0.30, -0.09) | -0.22 (-0.34, -0.11) |
|  | High | -0.35 (-0.46, -0.24) | -0.29 (-0.45, -0.14) | -0.25 (-0.41, -0.09) |
| Study participation RoB | Low | -0.18 (-0.26, -0.10) | -0.14 (-0.26, -0.02) | -0.11 (-0.23, -0.01) |
|  | High | -0.32 (-0.45, -0.18) | -0.38 (-0.64, -0.12) | -0.19 (-0.34, -0.04) |
| Confounding RoB | Low | -0.26 (-0.55, 0.02) | -0.06 (-1.05, 0.92) | -0.25 (-0.73, 0.22) |
|  | High | -0.43 (-0.67, -0.19) | -0.36 (-0.62, -0.10) | -0.42 (-3.81, 2.98) |
|  | | | | |
| *CI: Confidence Interval; SMD: Standardised Mean Difference* | | | | |

**Supplementary Table S7:** Population Attributable Fraction calculation

| **Step 1** | We converted the SMDs back into odds ratios, which was the most common original effect estimate measure (n=56) with a logistic approximation |
| --- | --- |
| **Step 2** | We then calculated the population attributable fraction (PAF) of 1% the 1.68 billion people who cannot afford a healthy diet in LMICs moving to ‘healthy diet’ status (1% = 16,800,000) |
| **Step 3** | We assume in this exercise that all those who could afford a healthy diet actually ate a healthy diet |
| **Result** | This translates to 41% lower odds of depression (OR 0.59, 95% CI 0.53 to 0.66), in turn with between 0.90–1.27 million less cases of depression per 1% change |
| **Notes** | These calculations are based on several strong assumptions with no assumption of causality, but are offered as an example of how modest effects can have large population-level implications. |

**Supplementary Figures**

**Supplementary Figure S1A-C:** Prediction intervals of the pooled effect comparing healthy diets to depression, anxiety and stress.

**A) Depression**

**
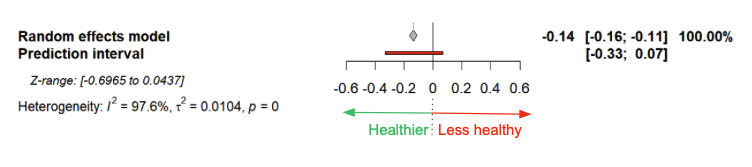
**

**B) Anxiety**

**
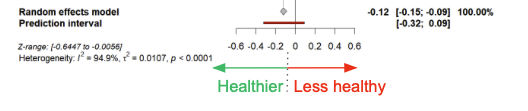
**

**C) Stress**

**
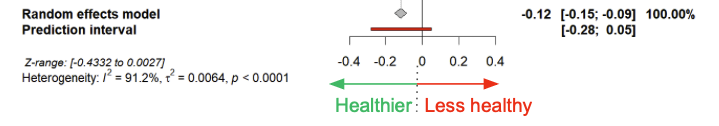
**

**Supplementary Figure S2:** Risk of bias (RoB) in longitudinal studies (n=9), including study attrition domain


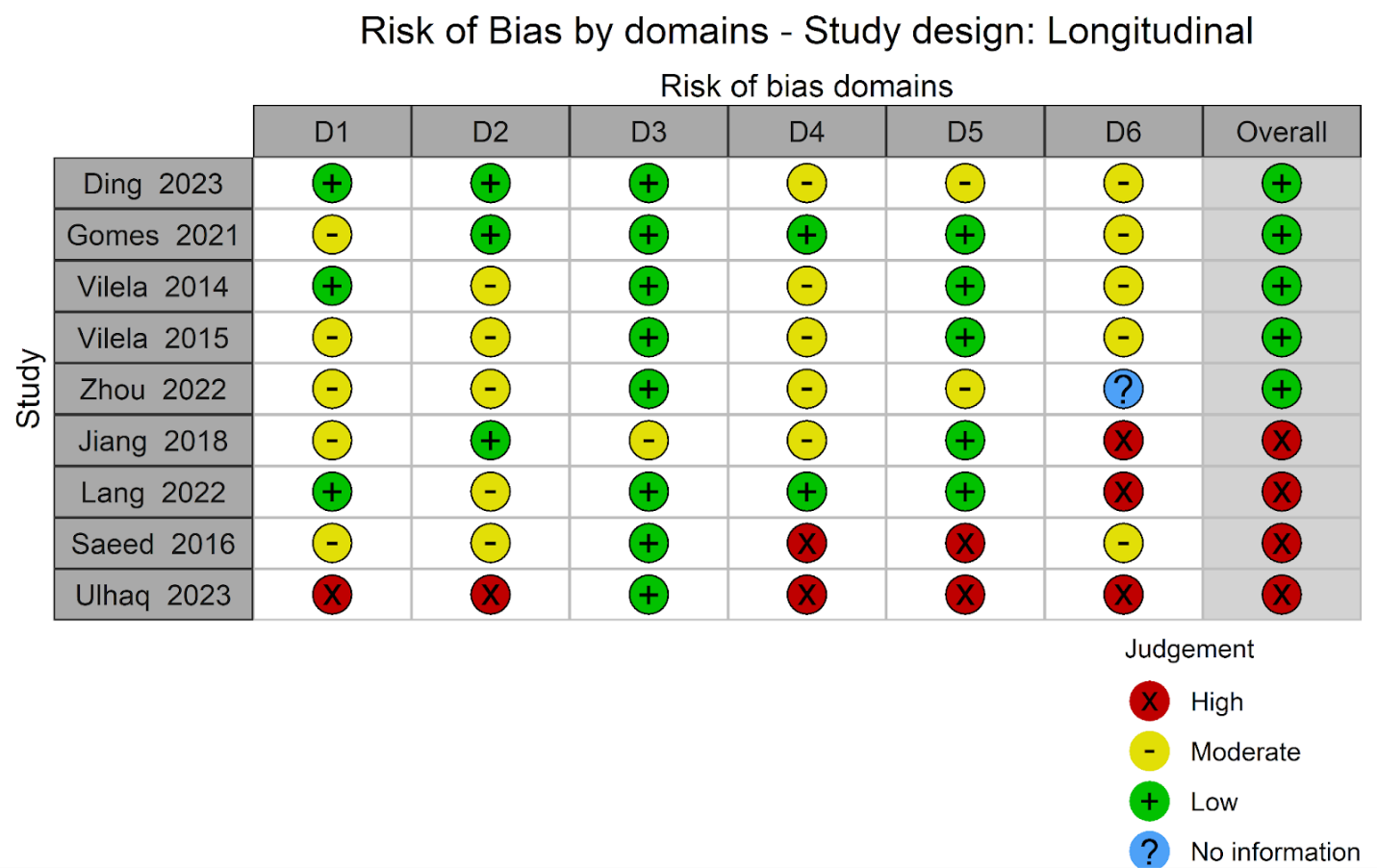


D1 = Bias due to study participation, D2 = Bias due to exposure measurement, D3 = Bias due to outcome measurement, D4= Bias due to confounding, D5 = Bias in statistical analysis and reporting, Overall = No high risk of bias in any domain, D6 = Bias due to attrition. Green = low RoB; Yellow = unclear RoB; Red = high RoB.

**Supplementary Figure S3:** Pooled effect estimates by dietary measure group.


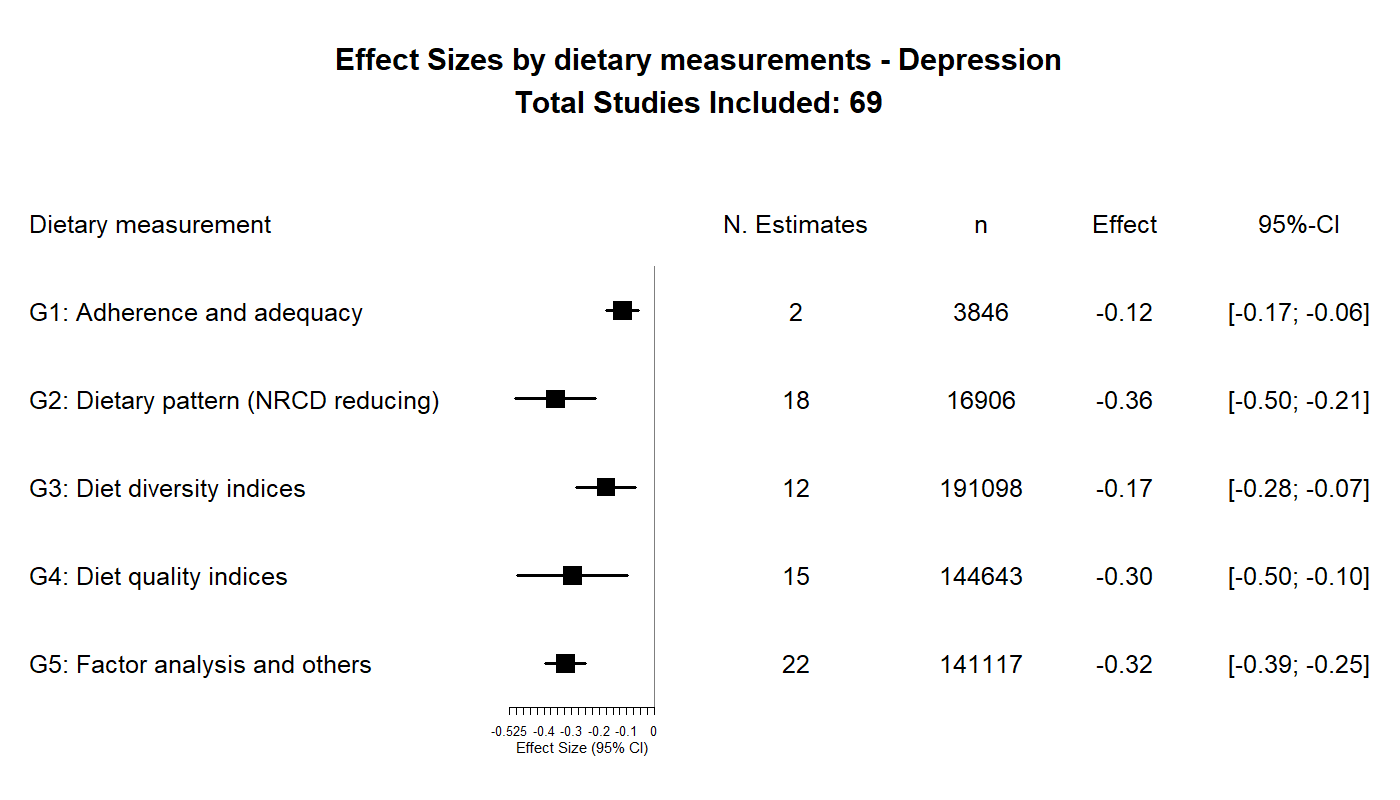

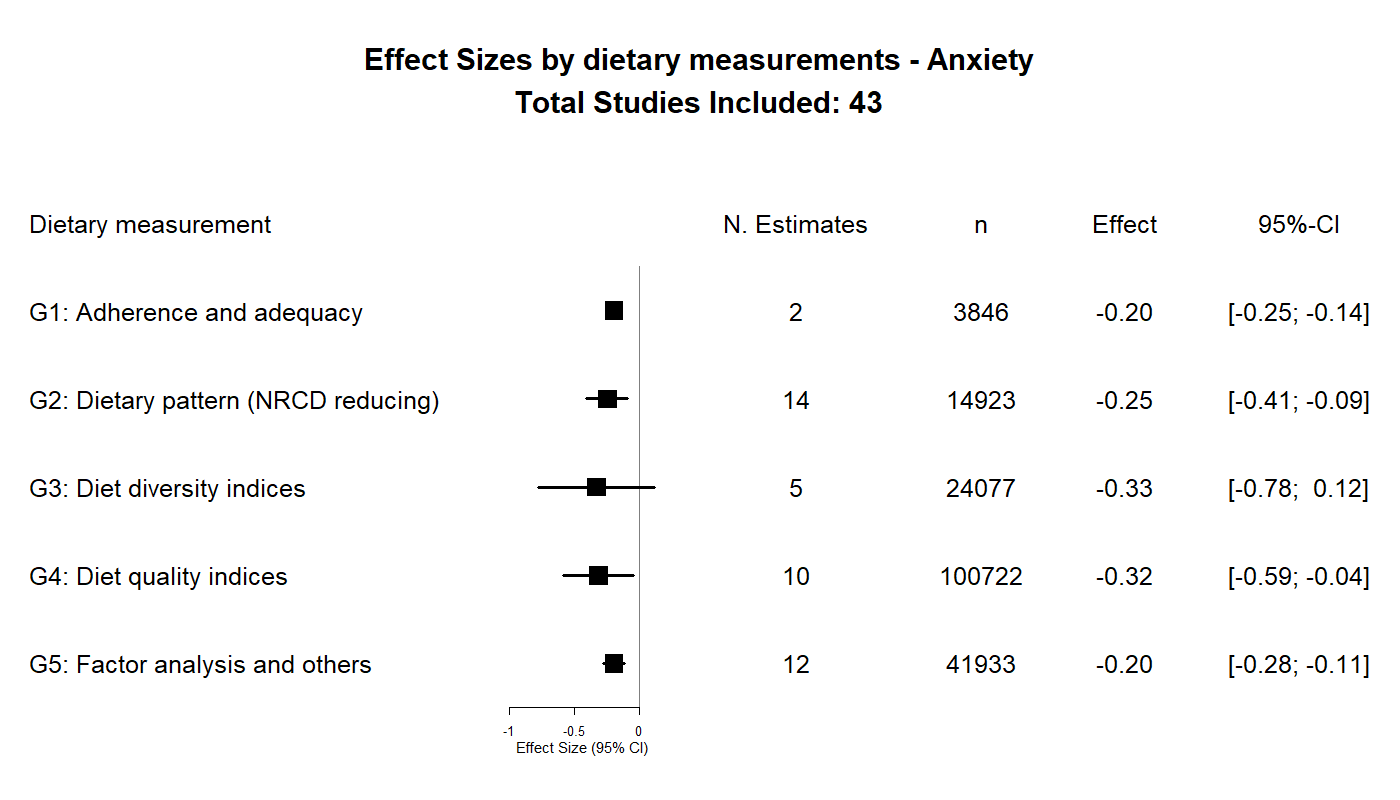


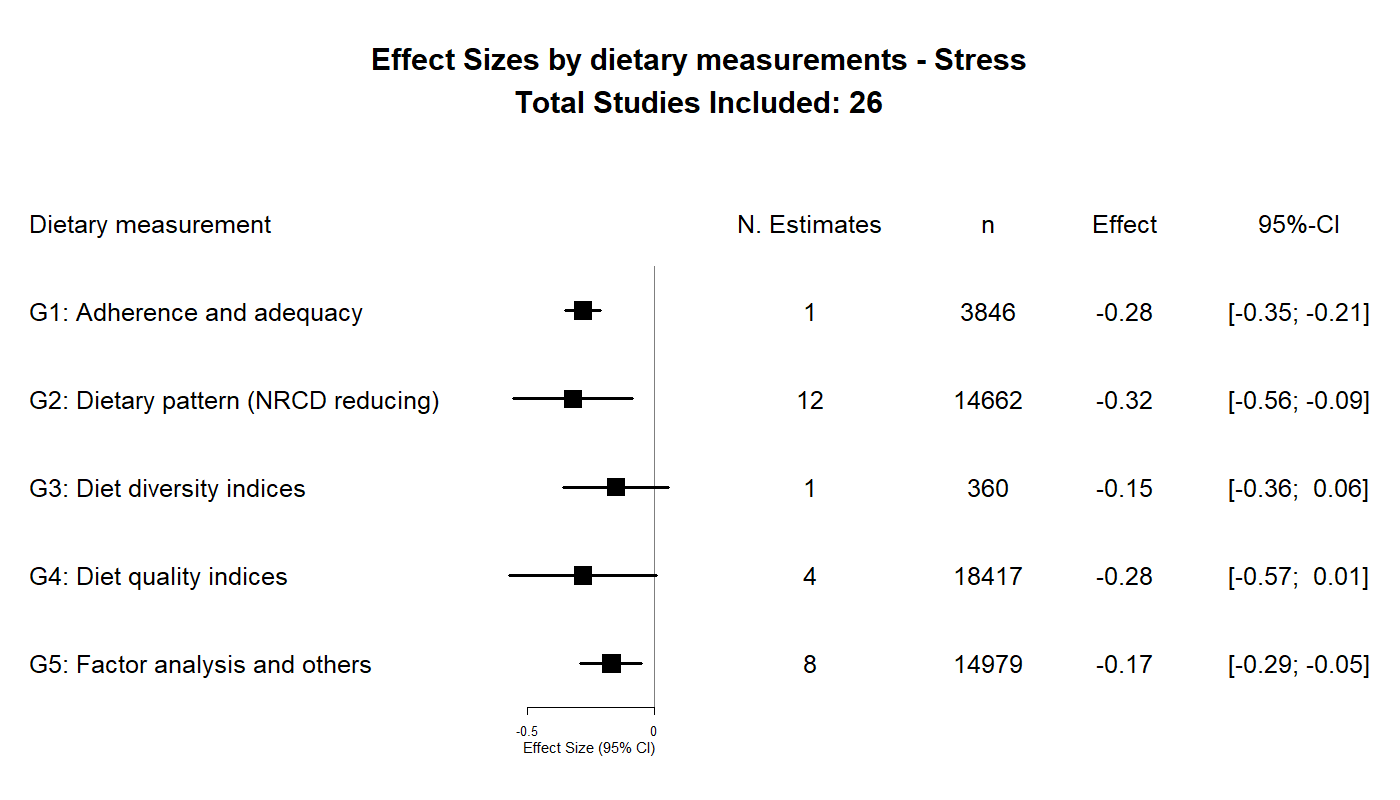


**Supplementary Figure S4: Sensitivity analysis to effect size indices choice**


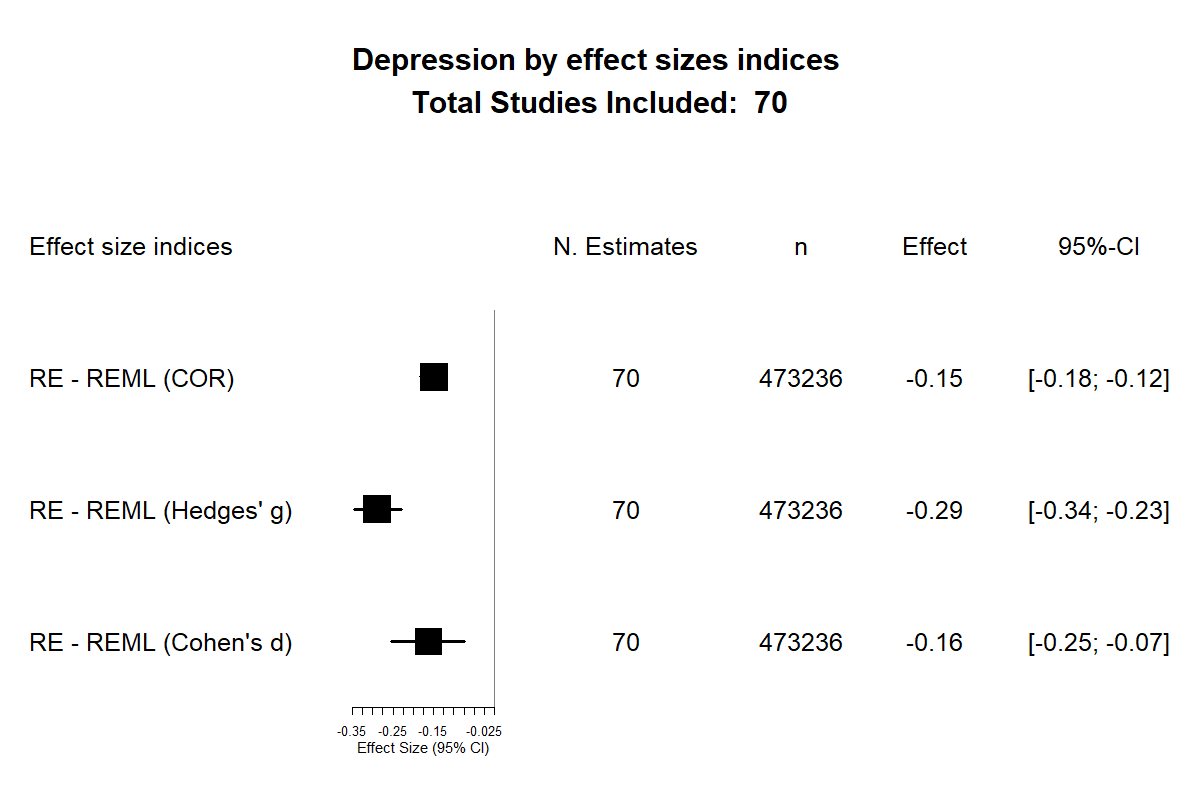

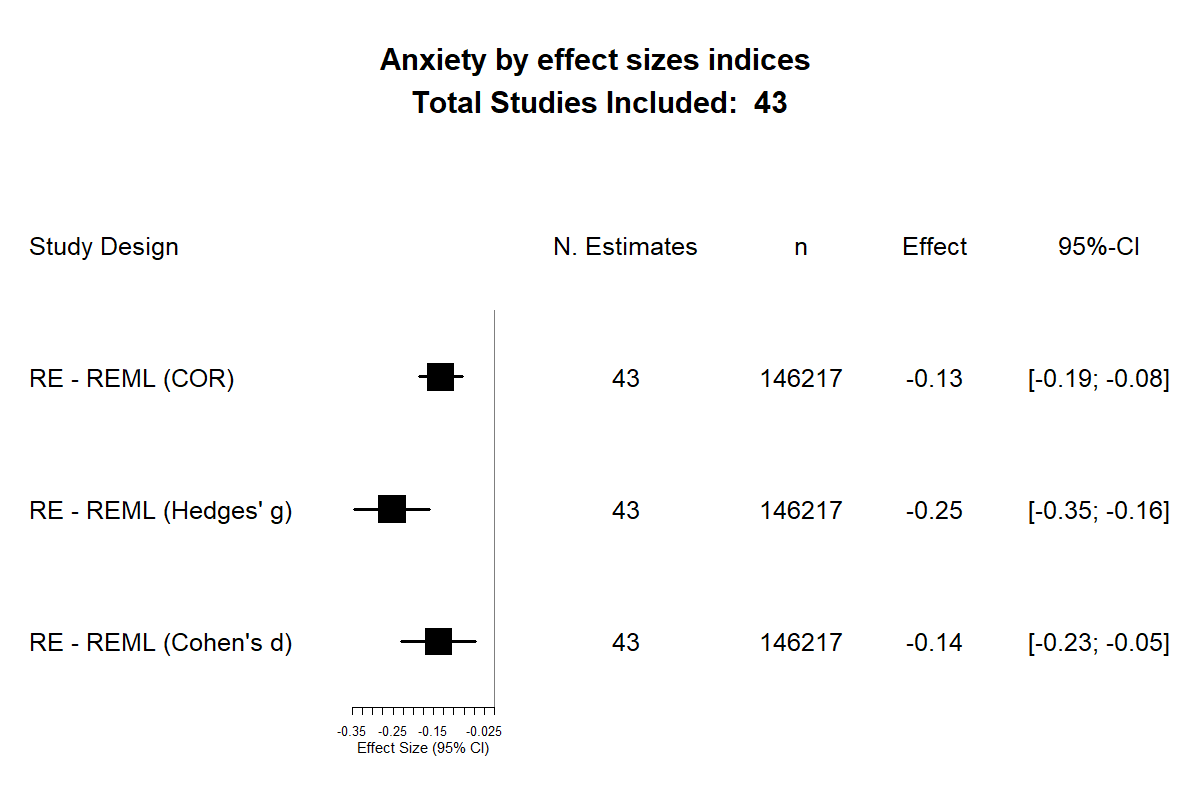


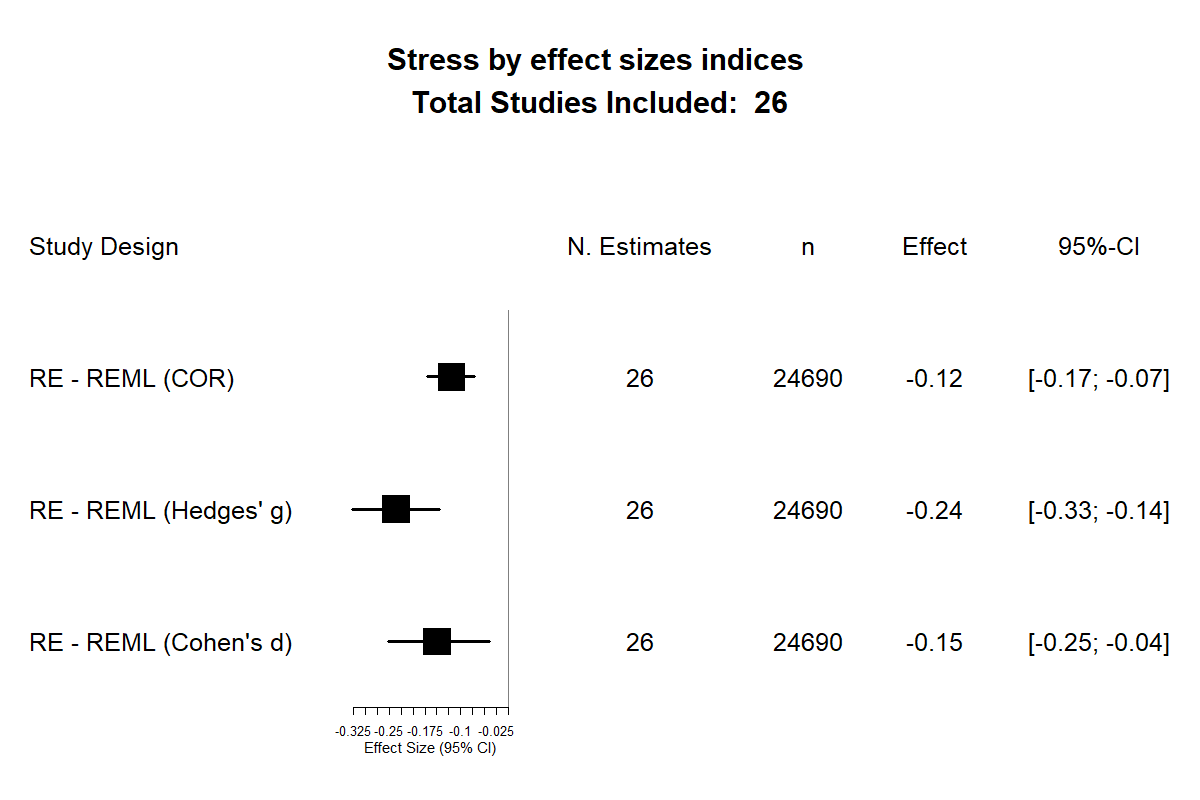

Supplement: Supplementary file 1 — Supplementary Material 1: Supplementary Tables. Supplementary Figures. [file 44263_2026_283_MOESM1_ESM.docx]
